# From Fixed-Frequency to Tunable: Advances in Acoustic Sensors for Physiological Acoustic Monitoring

**DOI:** 10.3390/s26092580

**Published:** 2026-04-22

**Authors:** Jiantao Wang, Chuting Liu, Peiyan Dong, Jiamiao Li, Kaiyuan Tan, Bo Li, Jianhua Zhou, Yancong Qiao

**Affiliations:** 1 School of Biomedical Engineering, Shenzhen Campus of Sun Yat-sen University, No. 66, Gongchang Road, Guangming District, Shenzhen 518107, China; wangjt56@mail2.sysu.edu.cn (J.W.); liucht9@mail2.sysu.edu.cn (C.L.); dongpy3@mail2.sysu.edu.cn (P.D.); lijm256@mail2.sysu.edu.cn (J.L.); tanky6@mail2.sysu.edu.cn (K.T.); zhoujh33@mail.sysu.edu.cn (J.Z.); 2 Key Laboratory of Sensing Technology and Biomedical Instruments of Guangdong Province, School of Biomedical Engineering, Sun Yat-sen University, Guangzhou 510275, China; 3 Cardiovascular Surgery, Seventh Affiliated Hospital of Sun Yat-sen University, Shenzhen 518107, China

**Keywords:** tunable-resonant frequency, acoustic sensor, physiological acoustic signal monitoring, frequency-selective amplification

## Abstract

Continuous, non-invasive cardiopulmonary monitoring is receiving increasing attention as population aging and chronic diseases rise. Acoustic sensing provides diagnostically relevant information with relatively simple hardware. Yet, physiological body sounds span heterogeneous and partially overlapping spectra and are highly susceptible to environmental noise and motion artifacts, which limit conventional stethoscopes and fixed-frequency sensors. Frequency-Tunable Acoustic Sensors (FTAS) offer a promising route toward frequency-selective amplification and adaptive interference suppression by matching their resonance to target signals, thereby potentially supporting multi-site monitoring and personalized diagnostics on a single platform. This review starts with an overview of physiological sound generation and the evolution of auscultation, then surveys mainstream medical acoustic transducers (piezoelectric, capacitive microelectromechanical systems (MEMS), piezoresistive and triboelectric) and their limitations in frequency selectivity. Resonance-tuning strategies are classified into three paradigms: electrical tuning, material-based tuning, and geometric reconfiguration, and their tuning ranges, response characteristics, and representative implementations are comparatively discussed. Finally, this review discusses the potential translational value of FTAS in physiological acoustic signal monitoring, particularly in cardiovascular and respiratory assessment, and emphasizes the remaining challenges, including the trade-off between sensitivity and selectivity, as well as long-term biocompatibility. At the same time, this review highlights their development prospects in customizable acoustic sensing platforms.

## 1. Introduction

The global aging population and the high prevalence of cardiovascular and respiratory diseases have created an urgent need for continuous and non-invasive monitoring of cardiopulmonary function [1,2]. Although conventional hospital-grade monitoring devices provide reliable measurements, their bulkiness, extensive wiring, and limited wearability restrict long-term use in daily life, thereby constraining early screening and remote health management [3]. In this context, acoustic sensing has emerged as a promising approach because of its non-invasive nature, rich physiological information, and relatively simple hardware requirements [4,5]. However, physiological acoustic signals exhibit complex and site-dependent frequency characteristics, while environmental noise and motion artifacts further hinder reliable signal extraction. Frequency-tunable acoustic sensors (FTAS) address these challenges by matching sensor responses to target signal frequencies, thereby enabling selective signal enhancement, noise suppression, and multi-site physiological monitoring [6,7].

**Figure 1:**
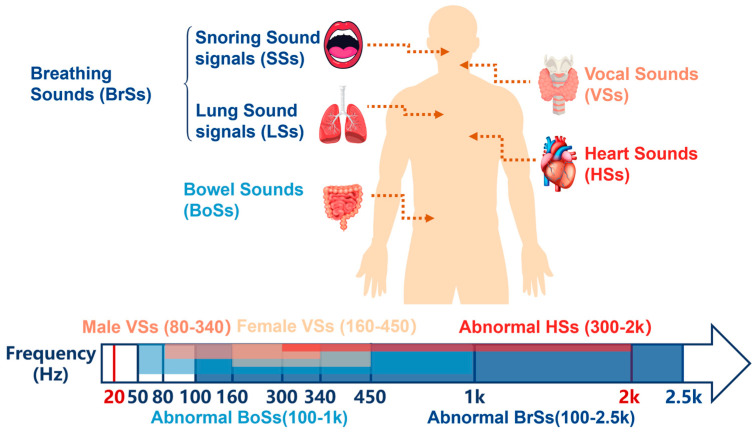
Different physiological signal frequency bands in different parts.

Physiological acoustic signals arise from elastic wave propagation induced by organ motion, including myocardial contraction, blood flow impact, airway turbulence, and gastrointestinal peristalsis [4]. Although weak in amplitude, these signals carry important information about organ function. As shown in Figure 1, their frequency distributions are highly heterogeneous and partially overlapping: Heart Sounds (HSs) mainly span 20–2000 Hz [8], Bowel Sounds (BoSs) 50–1000 Hz [2,9,10,11], abnormal Breathing Sounds (BrSs) 100–2500 Hz [12], and Vocal Sounds (VSs) 80–1200 Hz in males and 160–2000 Hz in females [13,14,15,16]. With suitable acoustic sensors, these signals can be acquired and analyzed for non-invasive assessment of cardiopulmonary and other organ functions. However, this spectral overlap makes fixed-frequency sensors prone to insufficient selectivity and signal interference, particularly in multi-organ monitoring and noisy environments.

Auscultation has been a fundamental method for cardiopulmonary screening since Laennec introduced the wooden stethoscope in 1816 [17]. Although conventional mechanical and electronic stethoscopes are effective for basic screening, their fixed acoustic characteristics limit selective amplification of pathological signals across different frequency bands, and their diagnostic performance often depends heavily on operator experience [18]. In addition, the spectral overlap between physiological acoustic signals and environmental noise makes interference suppression difficult for fixed-response devices, while static bandpass filters cannot adapt to changing signal characteristics in real time [19]. Current acoustic sensors, including piezoelectric, capacitive microelectromechanical systems (MEMS), piezoresistive, and triboelectric, offer diverse advantages in sensitivity, bandwidth, flexibility, and miniaturization [20]. However, most still operate at fixed resonant frequencies determined during fabrication, which limits multi-site auscultation and clinical versatility [21]. These limitations highlight the need for acoustic sensors capable of dynamically adjusting their operating frequency to match different physiological signals and clinical demands [4,19,20,22].

Existing reviews on wearable acoustic sensors and biomedical monitoring have mainly focused on physiological signal types, flexible and wearable device technologies, or the integration of sensing systems with data analysis and machine learning. In contrast, this review specifically focuses on FTAS for physiological acoustic monitoring. Its novelty lies in treating resonance tunability as the central theme, thereby providing a more focused perspective on the design principles, sensing characteristics, and biomedical significance of these devices beyond conventional summaries of wearable acoustic sensors.

This review focuses on tunable acoustic sensors for medical applications, linking physiological acoustic characteristics with sensor design and clinical use. Section 2 introduces the generation and spectral characteristics of physiological acoustic signals and briefly reviews the evolution of acoustic auscultation. Section 3 summarizes current acoustic sensors used in medical diagnostics, including piezoelectric, capacitive MEMS, piezoresistive, and triboelectric devices, with emphasis on their fixed-frequency limitations. Section 4 discusses the main tuning mechanisms and representative implementations of FTAS for physiological signal monitoring. Section 5 summarizes their key advantages, current challenges, and future directions for next-generation physiological acoustic monitoring systems. Overall, this review aims to provide a concise framework for understanding how tunable acoustic sensing may improve frequency-selective monitoring and support the development of more adaptive and clinically useful acoustic diagnostic technologies.

To further improve readability, Table 1 summarizes the main sensor categories covered in this review and their corresponding sections. This overview is intended to serve as a brief guide for readers.

## 2. Physiological Body Sounds

### 2.1. Generation Mechanisms and Spectral Characteristics of Physiological Acoustic Signals

**Figure 2:**
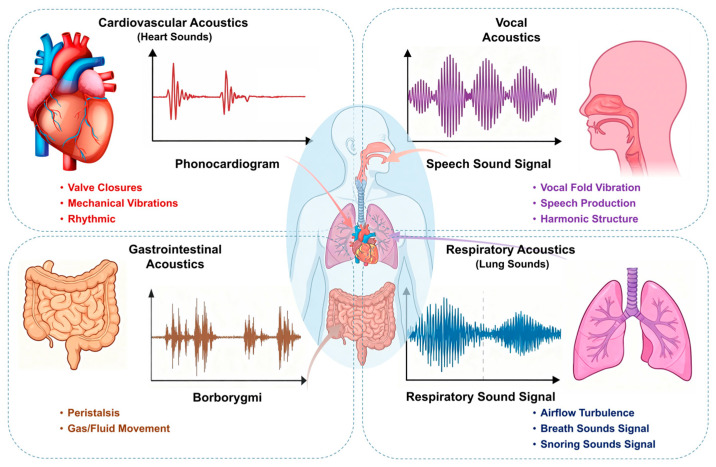
Organ schematic diagram and physiological acoustic signal waveform diagram.

Physiological acoustic signals arise from elastic wave propagation associated with the mechanical activity of internal organs [23]. Although these signals are often much weaker than ambient noise, they contain diagnostically relevant frequency components that reflect both normal physiological and pathological states [4,24,25]. Table 2 summarizes the representative frequency ranges of four types of physiological body sounds. Their heterogeneous spectral distributions across organ systems also provide the basis for frequency-selective acoustic monitoring (Figure 2) [26].

#### 2.1.1. Heart Sounds

HSs arise from myocardial motion and valve dynamics during the cardiac cycle [27,28]. The fundamental sounds S1 and S2 are mainly concentrated in the 20–150 Hz range [27,28,29,30,31,32]. Pathological murmurs caused by turbulent blood flow usually extend into higher frequencies, commonly within the 300–600 Hz range [33,34,35]. In addition, abnormal transient sounds, such as opening snaps and ejection clicks related to abnormal valve motion, may present at relatively higher frequencies [36,37]. These spectral differences make heart sounds an important target for frequency-selective sensing.

#### 2.1.2. Breathing Sounds

BrSs are generated by airflow within the airways and their interaction with surrounding tissues [38,39]. Normal vesicular breath sounds are mainly distributed in the 100–500 Hz range, with peak energy around 200–300 Hz [40,41]. Pathological respiratory sounds exhibit a broader spectral range, with wheezes typically appearing at 400–1000 Hz, crackles showing broadband characteristics, and stridor extending up to 2500 Hz under upper airway obstruction [38,42,43,44,45]. This wide spectral distribution highlights the need for adaptable respiratory sensing.

#### 2.1.3. Bowel Sounds

BoSs originate from peristaltic motion and fluid–gas interactions in the gastrointestinal tract [46]. Most bowel sounds fall within 50–300 Hz, while higher-frequency components up to 1000 Hz may arise from fluid movement and gas bubbling [10,47,48]. Pathological conditions such as bowel obstruction or acute gastroenteritis are often associated with increased sound intensity and components in the 300–800 Hz range, whereas paralytic ileus leads to sparse or absent bowel sounds [49,50]. Their intermittent and variable nature makes reliable detection more challenging.

#### 2.1.4. Vocal Sounds

VSs are generated by the vibration of the vocal folds during phonation [51]. The fundamental frequency is typically 80–180 Hz in adult males and 160–300 Hz in adult females [52,53]. Speech signals also contain higher-frequency harmonics and formants, with major energy distributed up to about 1200 Hz in males and 2000 Hz in females [53,54,55]. As a result, vocal signals present broader spectral requirements than many other physiological acoustic signals.

### 2.2. Evolution of Acoustic Auscultation

**Figure 3:**
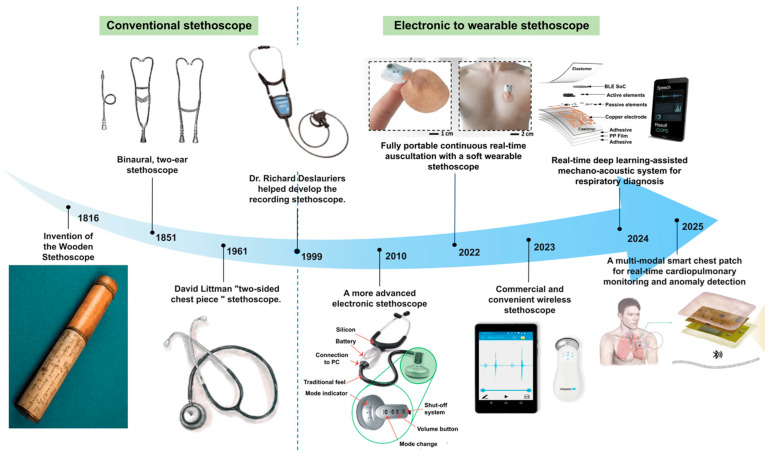
Timeline of acoustic stethoscope development. View representative acoustic stethoscope from 1816: The Wooden Stethoscope was first invented in 1816, David Littman’s “two-sided chest piece“ stethoscope was invented in 1961, and Dr. Richard Deslauriers helped develop the recording stethoscope in 1999 (Adapted from Ref. [64] under CC BY 4.0 license, 10.7759/cureus.75446). A more advanced electronic stethoscope was invented in 2010 (Adapted from Ref. [1] under CC BY 4.0 license, https://doi.org/10.1186/s12938-025-01457-7). Fully portable continuous real-time auscultation with a soft wearable stethoscope was made in 2022 (Adapted from Ref. [62] under CC BY 4.0 license, https://doi.org/10.1126/sciadv.abo5867). The commercial and convenient wireless stethoscope has emerged in 2023 (Adapted from Ref. [65] under CC BY 4.0 license, https://doi.org/10.3390/mi14112092). In 2024, a Real-time deep learning-assisted mechano-acoustic system for respiratory diagnosis was invented (Adapted from Ref. [66] under CC BY 4.0 license. (https://doi.org/10.1038/s41528-024-00355-7). A multi-modal smart chest patch for real-time cardiopulmonary monitoring and anomaly detection was invented in 2025 (Adapted from Ref. [67] under CC BY 4.0 license, https://doi.org/10.1007/s40843-025-3667-7).

The stethoscope has evolved from a simple mechanical auscultation tool to electronic, digital, and wearable systems (Figure 3). Since Laennec introduced the first wooden stethoscope in 1816, later developments such as binaural designs and diaphragm-based chest pieces progressively improved acoustic transmission and clinical usability [56,57,58,59,60]. More recently, electronic and wearable stethoscopes have enabled signal amplification, recording, wireless transmission, and real-time monitoring [61,62]. In parallel, other medical acoustic technologies, such as medical ultrasound and cochlear implants, have also advanced significantly, although they rely on active imaging or hearing-assistance mechanisms distinct from passive body sound sensing [4,63].

Despite these developments, most auscultation devices still rely on fixed acoustic response characteristics. This limitation motivates the development of frequency-adaptive sensing systems that can better match different physiological acoustic signals and improve diagnostic performance.

### 2.3. Diagnostic Value and Frequency Requirements Across Organ Systems

The diagnostic value of physiological acoustic monitoring depends strongly on the frequency characteristics of signals from different organ systems. Because cardiac, respiratory, gastrointestinal, and vocal sounds span distinct spectral ranges [30,45,68,69], conventional auscultation devices with fixed acoustic responses often cannot achieve optimal performance across all targets. In contrast, FTAS can better match the characteristic frequency bands of different physiological signals, enabling more selective sensing and improved suppression of out-of-band interference [70,71,72,73,74,75].

## 3. Fixed-Frequency Acoustic Sensors

The diverse spectral characteristics and low-amplitude nature of physiological body sounds, as delineated in Section 2, impose stringent demands on signal acquisition hardware. The fidelity of bio-acoustic monitoring fundamentally depends on the mechano-acoustic transduction interface between the sensor and human skin, which must efficiently couple weak vibrations from the skin surface and convert them into interpretable electrical signals with high signal-to-noise ratio (SNR).

**Figure 4:**
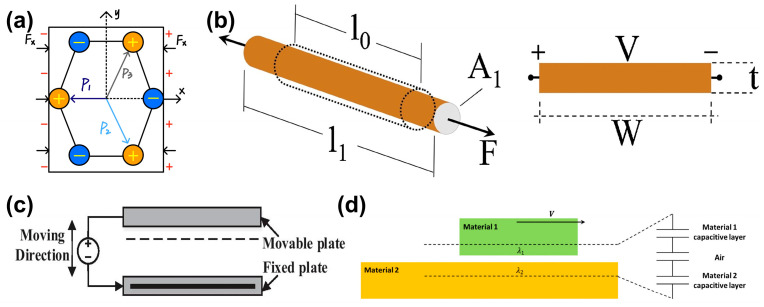
Schematic diagram of four sensing mechanisms. (**a**) Schematic diagram of the piezoelectric effect sensing mechanism. (**b**) Schematic diagram of the piezoresistive effect sensing mechanism. Adapted from Ref. [82] Copyright 2018, Elsevier. (https://doi.org/10.1016/j.sna.2018.07.006). (**c**) Schematic diagram of the capacitive MEMS effect sensing mechanism. Adapted from Ref. [83] under CC BY 4.0 license. (https://doi.org/10.1186/s11671-021-03481-7). (**d**) Schematic diagram of the sensing mechanism of the triboelectric effect. Adapted from Ref. [84] under CC BY 4.0 license. (https://doi.org/10.1007/s40544-018-0217-7).

Acoustic sensors have made significant progress in miniaturization and sensitivity over recent decades. Based on their transduction mechanisms, as shown in Figure 4, they can be grouped into four main types: piezoelectric, piezoresistive, capacitive (including MEMS), and triboelectric sensors [76,77,78]. However, despite differences in performance and fabrication complexity [79,80], most conventional sensors share a fixed frequency response determined during fabrication, highlighting the need for the frequency-tunable architectures discussed in Section 4 [81].

### 3.1. Piezoelectric Acoustic Sensors

Piezoelectric acoustic sensors convert sound-induced mechanical deformation into electrical signals through the direct piezoelectric effect [85].

**Figure 5:**
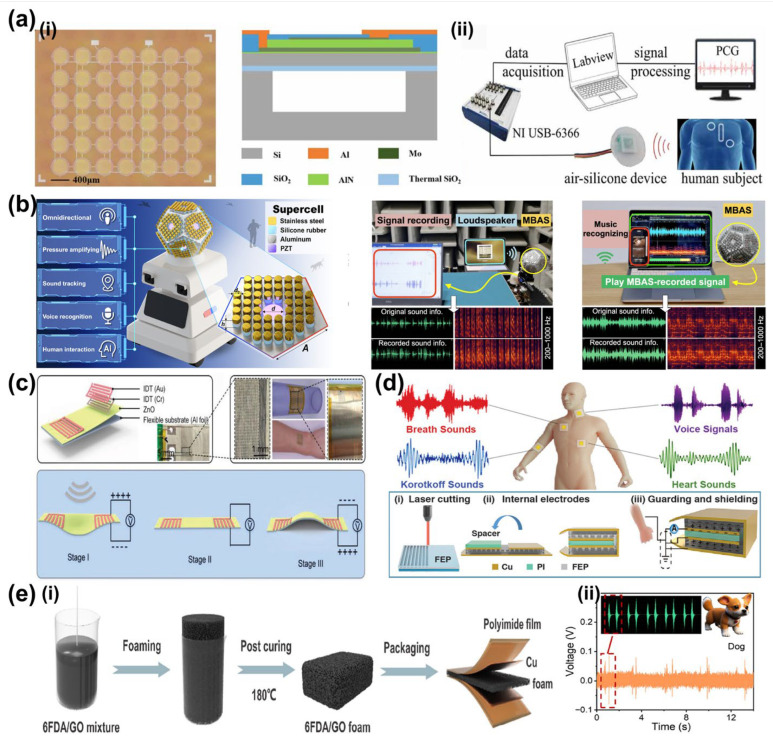
Application of Piezoelectric Sensors. (**a**) Wearable device based on a piezoelectric MEMS acoustic sensor. (**a**,**i**) The optical microscope images of the 6 by 7 array and the cross-sectional view of a single element. (**a**,**ii**) The measurement set-up of phonocardiography (PCG). (**b**) A wave-confining meta sphere beamforming acoustic sensor for superior human–machine voice interaction. Adapted from Ref. [93] under CC BY 4.0 license. (https://doi.org/10.1126/sciadv.adc9230). (**c**) Multifunctional and wearable patches based on flexible piezoelectric acoustics. Adapted from Ref. [90] under CC BY 4.0 license. (https://doi.org/10.1002/adfm.202209667). (**d**) Health monitoring via heart, breath, and Korotkoff sounds by wearable piezoelectric patches. Adapted from Ref. [91] under CC BY 4.0 license. (https://doi.org/10.1002/advs.202301180). (**e**) Ultrasensitive piezoelectric sensor based on PI foam for sound recognition and motion monitoring. (**e**,**i**) Principles of PI/GO foam reactions. (**e**,**ii**) Identification of the dog’s recording. Adapted from Ref. [92] Copyright @ 2025, American Chemical Society. (https://doi.org/10.1021/acsami.4c22301).

Owing to their high sensitivity and compact integration, they have been widely used in physiological acoustic monitoring. Representative materials include PZT, ZnO, AlN, PVDF, and their composites [86,87,88]. Qu et al. developed a wearable MEMS piezoelectric sensor for capturing heart sounds and speech in ambulatory environments (Figure 5a) [89]. Zhang et al. reported a flexible piezoelectric patch capable of distinguishing breath sounds and wheezes for respiratory diagnostics (Figure 5c) [90]. Han et al. introduced a flexible piezoelectric electret patch for simultaneous acquisition of heart sounds, Korotkoff tones, and crackles, enabling zoned valvular assessment (Figure 5d) [91]. Zhong et al. further developed a fluorinated polyimide foam piezoelectric sensor for long-term bowel sound monitoring (Figure 5e) [92]. These studies demonstrate the broad applicability of piezoelectric sensors in multi-organ auscultation, although their resonance characteristics remain constrained by fixed structural design.

### 3.2. Capacitive Acoustic Sensors

Capacitive acoustic sensors detect sound through capacitance changes caused by diaphragm vibration under acoustic pressure [94]. In a parallel-plate configuration, the capacitance is defined as

(1) $$C=\frac{\varepsilon A}{d}$$

where *A* is the diaphragm–backplate overlap area, *d* is the instantaneous separation, and *ε* is the permittivity of the dielectric medium [95]. Owing to their low mass and high compliance, capacitive diaphragms provide high sensitivity at audio frequencies relevant to body sounds, although their resonance frequency remains fixed after fabrication [96].

**Figure 6:**
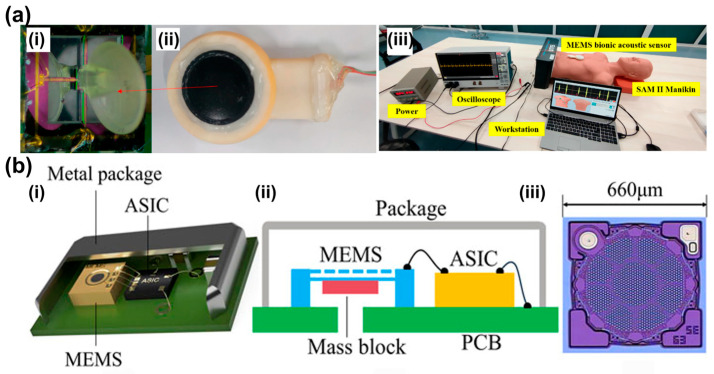
The application of capacitive acoustic sensors. (**a**) Design of a novel medical acoustic sensor based on MEMS bionic fish ear structure. (**a**,**i**) Integrated diagram of the fish ear structures sensor. (**a**,**ii**) The sensor probe encapsulation. (**a**,**iii**) Heart sound detection test. Adapted from Ref. [101] under CC BY 4.0 license. (https://doi.org/10.3390/mi13020163). (**b**) Design and implementation of an acoustic-vibration capacitive MEMS microphone. (**b**,**i**) The structure of the sensor. (**b**,**ii**) The mass block attached to the microphone. (**b**,**iii**) The dimension and geometry of the backplate structure. Adapted from Ref. [102] under CC BY 4.0 license. (https://doi.org/10.1063/5.0090687).

Typical devices are based on silicon MEMS structures, while ultrathin materials such as graphene have also been explored to improve sensitivity and compliance [97,98,99]. Capacitive and MEMS acoustic sensors have been applied in structural monitoring, speech acquisition, and biomedical auscultation because of their simple architecture and rapid response. Zhang et al. reported a silicon MEMS capacitive acoustic emission sensor for structural health monitoring, capable of detecting both continuous and burst-type emissions [100]. In biomedical monitoring, Zhou et al. proposed a biomimetic fish-ear-inspired MEMS auscultation sensor that enhanced cardiac sound acquisition through mechanical filtering (Figure 6a) [101]. Duanmu et al. designed a humidity- and dust-resistant vibration–acoustic MEMS microphone for low-frequency heart sound monitoring in wearable scenarios (Figure 6b) [102]. Li et al. further highlighted the diagnostic potential of capacitive micromachined ultrasonic transducers for hybrid acoustic monitoring and ultrasonic imaging [103]. Together, these studies show that capacitive acoustic sensors provide versatile platforms for physiological monitoring, while their performance is still constrained by fixed diaphragm resonance.

### 3.3. Piezoresistive Acoustic Sensors

Piezoresistive acoustic sensors detect sound through resistance changes induced by mechanical deformation [104,105,106]. Their performance depends strongly on the sensing materials and structural design, with silicon-based piezoresistors and emerging carbon-based materials such as Laser-Induced Graphene (LIG), reduced Graphene Oxide (rGO), and Carbon Nanotubes (CNTs) being widely investigated for acoustic sensing [107,108,109,110,111,112,113]. Similar to other conventional acoustic sensors, their frequency response is determined by structural resonance and remains fixed after fabrication.

**Figure 7:**
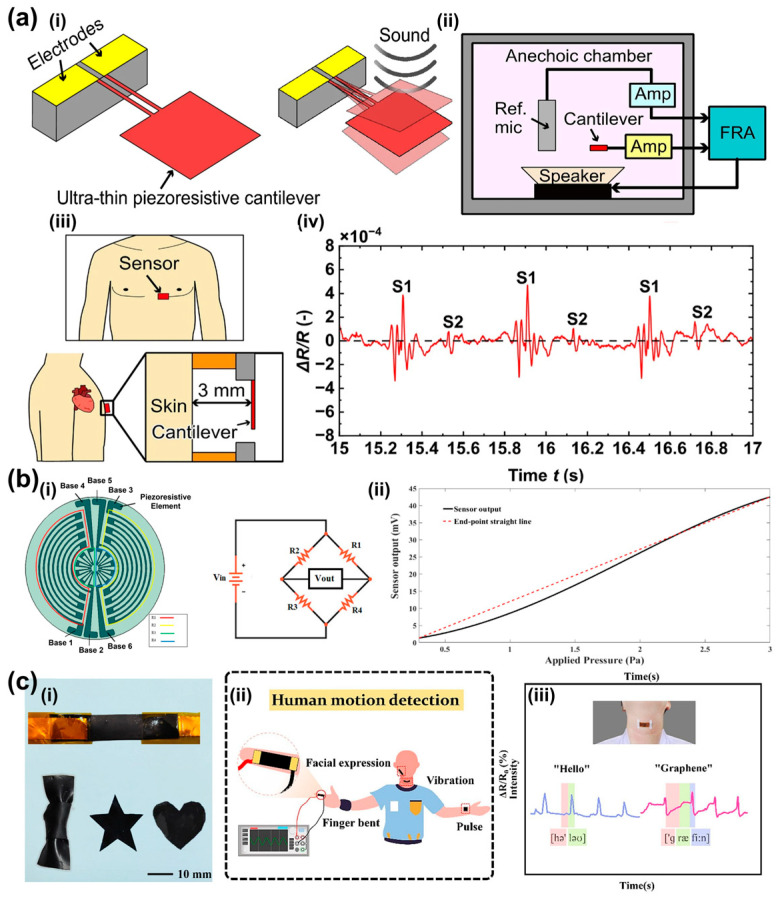
Piezoresistive sensors applications. (**a**) Highly sensitive low-frequency-detectable acoustic sensor using a piezoresistive cantilever. (**a**,**i**) Conceptual illustration of the proposed microphone using a piezoresistive cantilever. (**a**,**ii**) Conceptual design of the experimental setup to evaluate the performance of the cantilever. (**a**,**iii**) Conceptual schematic of the proposed sensor for measuring heart sound. (**a**,**iv**) A zoomed-in view of the recorded heart sound in the duration of 15–17 s. Adapted from Ref. [115] under CC BY 4.0 license. (https://doi.org/10.1038/s41598-023-33568-3). (**b**) Design and fabrication of a LEG-based stretchable piezoresistive acoustic pressure sensor for ultra-low pressures. (**b**,**i**) Structure of the presented sensor and Wheatstone bridge formed by four resistors on the sensor. (**b**,**ii**) Measured PS output. Adapted from Ref. [107] under CC BY 4.0 license. (https://doi.org/10.1038/s41598-025-97091-3). (**c**) PDMS-based conductive elastomeric composite with 3D reduced graphene oxide conductive network for flexible strain sensor. (**c**,**i**) Fabrication process of the rGO/PDMS film. (**c**,**ii**) Schematic diagram of human motion detection. (**c**,**iii**) The sensor was fixed on the throat to monitor the relative resistance change when the volunteer says “Hello” and “Graphene”. Adapted from Ref. [116] Copyright 2022, Elsevier. (https://doi.org/10.1016/j.compositesa.2022.107113).

Piezoresistive sensors have been applied in diverse acoustic monitoring scenarios because of their high sensitivity to subtle vibrations. Sun et al. developed a bio-inspired graphite/PDMS composite sensor for monitoring phonation when attached to the larynx [114]. Okamoto et al. reported an ultra-sensitive piezoresistive cantilever sensor capable of resolving low-frequency heart sounds for high-precision diagnostics (Figure 7a) [115]. Shiri et al. designed a stretchable LIG acoustic pressure sensor for ultra-low-pressure detection, with potential applications in underwater noise detection and environmental monitoring (Figure 7b) [107]. Wang et al. further developed an rGO/Kapton vector acoustic sensor for detecting finger movements, facial muscle activity, voice recognition, and other physiological signals (Figure 7c) [116]. These studies demonstrate the versatility of piezoresistive platforms in acoustic monitoring, although their fixed structural resonance still limits broadband adaptation across different physiological signals.

### 3.4. Triboelectric Acoustic Sensors

Triboelectric acoustic sensors convert acoustic vibrations into electrical signals through triboelectrification and electrostatic induction, enabling self-powered acoustic sensing [117,118]. Their performance depends strongly on the contact materials and device structure, with polymers such as Fluorinated Ethylene Propylene (FEP), Polytetrafluoroethylene (PTFE), Polydimethylsiloxane (PDMS), and Polyvinylidene Fluoride (PVDF)-based composites widely used to improve charge output and sensitivity [71,119,120,121,122,123,124,125,126,127,128,129].

**Figure 8:**
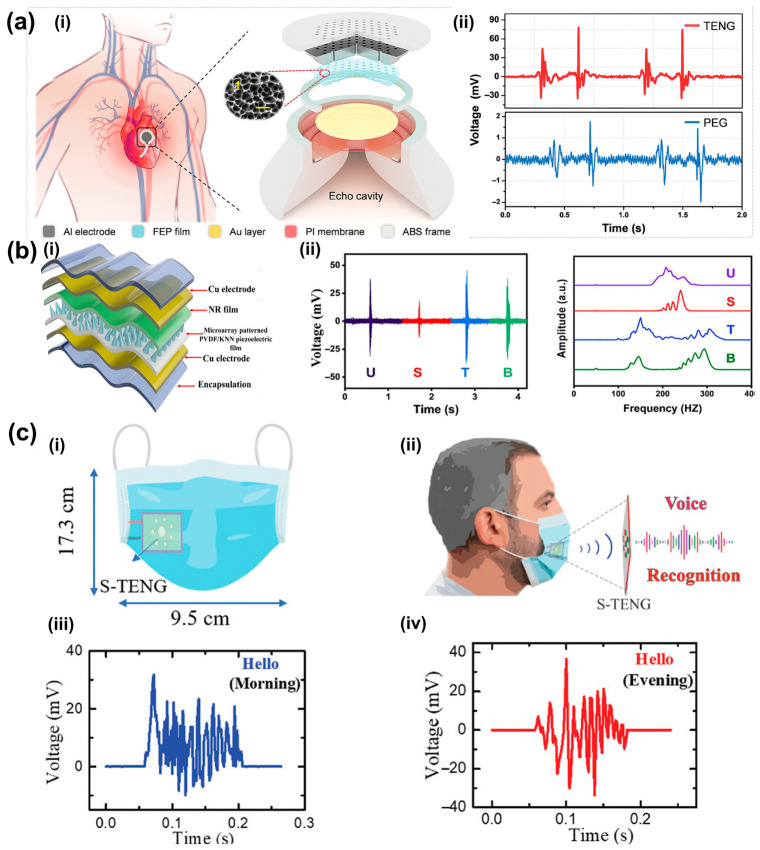
Triboelectric acoustic sensors applications. (**a**) Acoustically Enhanced Triboelectric Stethoscope for Ultrasensitive Cardiac Sounds Sensing. (**a**,**i**) Schematic illustration of cardiac sounds sensing using the triboelectric stethoscope and exploded view diagram of the overall design structure. Inset Figure: The surface morphology of the tribolayer (scale bar: 1 µm). (**a**,**ii**) Output electrical signal comparison of triboelectric and piezoelectric stethoscopes for real body test. Adapted from Ref. [131], Copyright 2024 Wiley-VCH GmbH. (https://doi.org/10.1002/adma.202401508). (**b**) Design of self-powered triboelectric–piezoelectric hybrid acoustic sensor. (**b**,**i**) Schematic diagram of the structure for the triboelectric-piezoelectric hybrid acoustic sensor (TPAS). (**b**,**ii**) Voltage signals of distinct phonemes (U, S, T, B) and FFT analysis results of different letter signals. Adapted from Ref. [133], Copyright 2025 Wiley-VCH GmbH. (https://doi.org/10.1002/adfm.202514004). (**c**) Design of a surface-potential-tuned single-material triboelectric acoustic sensor using electrospun nylon nanofibers. (**c**,**i**) The schematic of the facemask with the integration of single-TENG (S-TENG). (**c**,**ii**) Schematic description of the acoustic data recording with a facemask. (**c**,**iii**) Acoustic signal of the author’s voice of pronouncing “Hello” in the early morning measured by S-TENG. (**c**,**iv**) Acoustic sigSnal of the author’s voice of pronouncing “Hello” in the evening measured by S-TENG. Adapted from Ref. [134], Copyright 2022 Wiley-VCH GmbH. (https://doi.org/10.1002/smll.202201331).

Since the seminal introduction of the Triboelectric Nanogenerator (TENG) concept by Niu et al. in 2015 [130], self-powered acoustic sensing has evolved rapidly. Triboelectric acoustic sensors have attracted increasing attention for self-powered physiological monitoring. Hui et al. designed a triboelectric stethoscope for heart sound detection, achieving high diagnostic accuracy for cardiac conditions when combined with machine learning (Figure 8a) [131]. Xia et al. developed a flexible acoustic triboelectric sensor with ultra-wide bandwidth, supporting applications in laryngeal health monitoring, voice interaction, and ultrasonic diagnostics [132]. Shahbaz et al. reported a triboelectric–piezoelectric hybrid acoustic sensor for voice classification, with potential applications in laryngeal or respiratory assessment (Figure 8b) [133]. Babu et al. further developed a single-material triboelectric acoustic sensor capable of distinguishing subtle changes in vocal cord condition, indicating promise for diagnosing laryngeal pathologies (Figure 8c) [134]. Recent reviews have further summarized the use of triboelectric sensors as epidermal or textile stethoscopes and respiratory microphones for monitoring heart sounds, lung sounds, and airflow noise in a self-powered manner [135,136].

Despite these achievements, the reliance on fixed cavity dimensions and membrane properties in most acoustic designs still poses challenges for adaptive tuning across specific narrow physiological bands.

## 4. Frequency-Tunable Acoustic Sensors and Designs

Resonant frequency is the frequency at which a system exhibits the maximum oscillation amplitude under external excitation [137]. For most acoustic sensors, including piezoelectric, MEMS, and triboelectric devices, the dynamic behavior can be described by a mass–spring–damper model [73,138]. In an ideal case, the resonance frequency is determined by the equivalent stiffness and mass:

(2) $$f_{r}=\frac{1}{2\pi }\sqrt{\frac{k}{m}}$$

At resonance, the system achieves efficient energy transfer from the acoustic source to the sensing element, leading to enhanced sensitivity and SNR [75,139]. This feature is particularly important for detecting weak physiological acoustic signals.

In this section, frequency-tuning mechanisms are classified according to their underlying physical principles [74,140], including electrical tuning, material/physical property tuning, and geometric reconfiguration [72,140,141]. Their representative designs and potential relevance to medical diagnostics are discussed in the following subsections.

### 4.1. Electrical Tuning Mechanisms and Architectures

Electrical tuning is a highly integrable and precise strategy for modulating the resonance frequency of acoustic devices [142,143]. Unlike mechanical approaches, electrical methods use voltage or current to alter the effective stiffness, material properties, or equivalent circuit parameters of the system [144,145,146]. Based on the underlying mechanism, current electrical tuning strategies can be broadly divided into three categories [147].

#### 4.1.1. Electrostatic and MEMS Actuation Strategies

In MEMS devices, applying a DC bias voltage generates an electrostatic force that induces stress or deformation in the resonant membrane or beam, thereby modifying its effective stiffness or geometry and shifting the resonance frequency [148].

Electrostatic actuation is widely used in MEMS frequency tuning because of its compatibility with standard fabrication processes. Pang et al. first integrated a Film Bulk Acoustic Resonator (FBAR) with an electrostatic MEMS actuator in 2007, achieving a tuning range of 1.47% while maintaining a high quality factor, thus demonstrating the feasibility of low-power frequency tuning in high-frequency acoustic filters [148].

**Figure 9:**
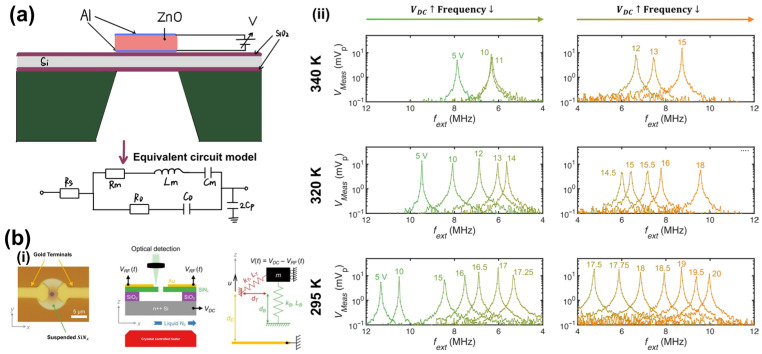
Electrical Tuning applications. (**a**) Schematic diagram of the electrical frequency-tuning mechanism in an acoustic resonator. (**b**) Ultra-wide tuning capability in MEMS drumhead resonators. (**b**,**i**) Design and geometry of the micro-drumhead resonator. (**b**,**ii**) Electrostatic and thermoelastic tunability of the resonator’s linear dynamic response. Experimentally measured frequency response of the resonator at temperatures of 340 K, 320 K, and 295 K for increasing, whose values label the respective response. Adapted from Ref. [149] Copyright 2023, Elsevier. (https://doi.org/10.1016/j.ymssp.2023.110331).

More recently, Kanj et al. reported MEMS drumhead resonators with an ultra-wide tuning range exceeding 230% by combining electrostatic control with thermal modulation (Figure 9b), highlighting the potential of hybrid tuning strategies for versatile acoustic signal processing [149].

#### 4.1.2. Piezoelectric Modulation: Stress and Modulus Control

For piezoelectric materials, external electric fields can tune the resonance frequency by modulating stress or elastic properties [150].

Stress-Induced Tuning: Nastro et al. utilized the inverse piezoelectric effect to tune piezoelectric MEMS acoustic transducers in 2019. By applying a DC bias voltage to the piezoelectric layer, they controlled the in-plane stress of the diaphragm. Their experiments demonstrated that within a bias range of ±8 V, the resonance frequency could be tuned by ±70 Hz, with a sensitivity of approximately 8.7 ± 0.5 Hz/V in transmission mode and 7.8 ± 0.9 Hz/V in reception mode. This technique is particularly valuable for electrically matching the series and parallel resonance frequencies, thereby optimizing the transducer’s performance [151].

**Figure 10:**
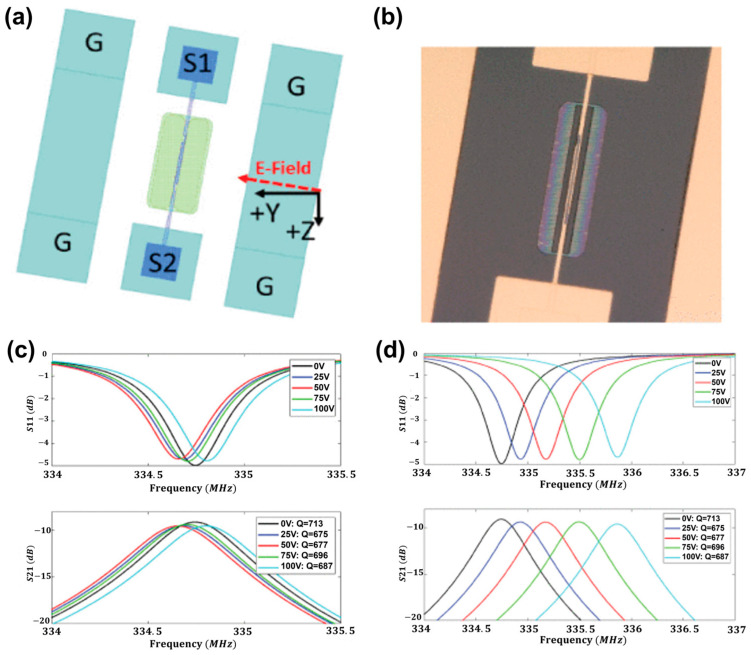
Solid-state tuning behavior via dc bias for SH_0_ mode. (**a**) A single resonator consisted of a pair of interdigital fingers. *λ* = 8 μm. The bias field was oriented −10° from the +Y-axis. (**b**) An optical micrograph image of a single resonator. (**c**) DC polarity was S1(−V)/S2(+V) with a negligible tuning effect. (**d**) DC polarity was S1(+V)/S2(−V). Adapted from Ref. [150] Copyright 2019, IEEE. (https://doi.org/10.1109/TUFFC.2019.2944174).

Modulus-Based Tuning: Beyond simple stress generation, strong electric fields can alter the material’s stiffness. Branch et al. exploited the piezoelectric nonlinearity of LiNbO_3_ thin plates in 2019 (Figure 10). They directly modulated the elastic modulus of the material and achieved a tuning of 0.4% (6 kHz/V at 335 MHz) by applying a DC bias field. This approach provides a low-loss, material-intrinsic tuning mechanism for broadband filters [150].

Expanding into the domain of traveling waves, Shao et al. achieved a breakthrough in controlling GHz-frequency acoustic waves in LiNbO_3_ waveguides in 2021. By leveraging the acoustoelectric effect to regulate the elastic modulus, they realized the first programmable modulation of the phase, frequency, and amplitude of traveling acoustic waves. This work lays the foundation for future Phonon Integrated Circuits (PICs) and quantum acoustic applications [74].

#### 4.1.3. Programmable Circuit Tuning

Circuit-Based Tuning: By integrating external variable components (varactors, inductors) or active feedback circuits (e.g., MOSFET switches) with the acoustic resonator, the system’s equivalent electrical parameters are modified, effectively “pulling” the resonance frequency of the coupled system [152,153,154].

For lower-frequency acoustic systems, circuit-level manipulation offers flexibility without requiring material deformation. Zhang et al. proposed an electrically tunable Helmholtz resonator based on a programmable MOSFET circuit. By digitally switching the connection states of circuit branches, they altered the equivalent acoustic parameters, achieving a tuning range of one octave in experiments. Theoretically, this method allows for infinite tuning ranges, offering a robust solution for programmable acoustic systems where physical geometry cannot be easily altered [155].

#### 4.1.4. Relevance to Medical Sensing and Diagnostics

The transition from static acoustic components to electrically programmable architectures mark a pivotal advancement for biomedical applications, particularly in the domains of implantable devices and high-frequency diagnostics. The ability to dynamically tune resonance frequencies allows medical sensors to adapt to complex physiological environments and optimize signal acquisition in real-time.

Recent breakthroughs in Surface Acoustic Wave (SAW) technology have significantly expanded the capabilities of implantable medical electronics. Arora et al. developed a SAW resonator based on magnetostrictive Ni-Mn-In and high-orientation piezoelectric lead magnesium niobate–lead titanate (PMN-PT) layers. By applying a DC bias voltage, they achieved a remarkable resonance frequency tuning of 8.9% at 2.52 GHz, alongside high sensitivity and Quality Factor. The authors explicitly highlighted that this dual-field (electric and magnetic) tunable architecture offers a new paradigm for biomedical implants, enabling devices that can reconfigure their operating frequency post-implantation to match varying tissue impedances or communication protocols [156].

For internal physiological monitoring, miniaturization and high-frequency precision are paramount. Sun et al. reported a high-frequency electrically tunable FBAR utilizing 2D α-In_2_Se_3_ flakes. Through control voltage modulation, the device achieved a precise frequency shift of 26 MHz. Although originating from Radio Frequency (RF) communication research, its high-frequency operation and electrical tunability provide the theoretical foundation and technical reserves for next-generation implantable acoustic sensors and medical ultrasound signal monitoring, where precise frequency selection allows for better resolution and tissue penetration depth control [157].

The integration of electrical tuning with biological recognition layers represents the future of non-invasive diagnostics. In a comprehensive review, Mujahid et al. emphasized that electrically tunable SAW resonators are uniquely suited for high-sensitivity detection of human acoustic signals. By combining electrical tuning with specific bio-recognition layers, these devices can isolate specific pathological signals from background noise. The authors posit that future high-frequency tunable SAW devices will revolutionize medical acoustic signal monitoring and non-invasive diagnostics, offering a versatile platform for detecting biomarkers and physiological anomalies with unprecedented accuracy [158].

In summary, electrical tuning mechanisms ranging from SAW to FBAR technologies are solving the critical challenge of “frequency rigidity” in medical sensors, paving the way for adaptive, high-precision diagnostic systems [140].

### 4.2. Material-Based and Physical Property Tuning

Material-based tuning strategies focus on directly modifying the intrinsic physical properties of the acoustic resonator, specifically its effective mass, elastic modulus, or acoustic impedance. By leveraging chemical infiltration, magnetic field-induced mechanical changes, physical deformation, or composite material engineering, these methods provide robust solutions for diverse environmental and operational conditions.

#### 4.2.1. Chemical Infiltration and Adsorption

The resonance frequency of nanomechanical systems is highly sensitive to mass loading and stiffness variations. Mechanism: In mesoporous materials, the infiltration of gas or liquid molecules into the pore network significantly alters the material’s average density and sonic velocity. This process, often reversible, allows for frequency tuning based on the extent of chemical adsorption or capillary condensation.

Oliveira et al. exploited this mechanism by fabricating nanomechanical resonators using mesoporous silica (SiO_2_) and titania (TiO_2_) thin films. By infiltrating these porous matrices with different chemical substances, they directly modified the mass density and elastic properties of the films. The study demonstrated a remarkable 60% tuning range of the acoustic resonance frequency, with the device exhibiting a linear response to environmental humidity changes. This approach establishes a new platform for reconfigurable nano-acoustic devices and highly sensitive environmental sensors where the tuning mechanism itself serves as the sensing modality [159].

#### 4.2.2. Magnetic Field-Induced Property Modulation

Magnetic fields offer a non-contact method to manipulate the mechanical properties of magnetostrictive or superparamagnetic materials [156]. Mechanism: When subjected to an external magnetic field, these materials undergo magnetostriction (geometric deformation) or exhibit the Delta-E effect, where the Young’s modulus changes as a function of magnetic field intensity. This directly alters the stiffness of the resonator, shifting its natural frequency.

**Figure 11:**
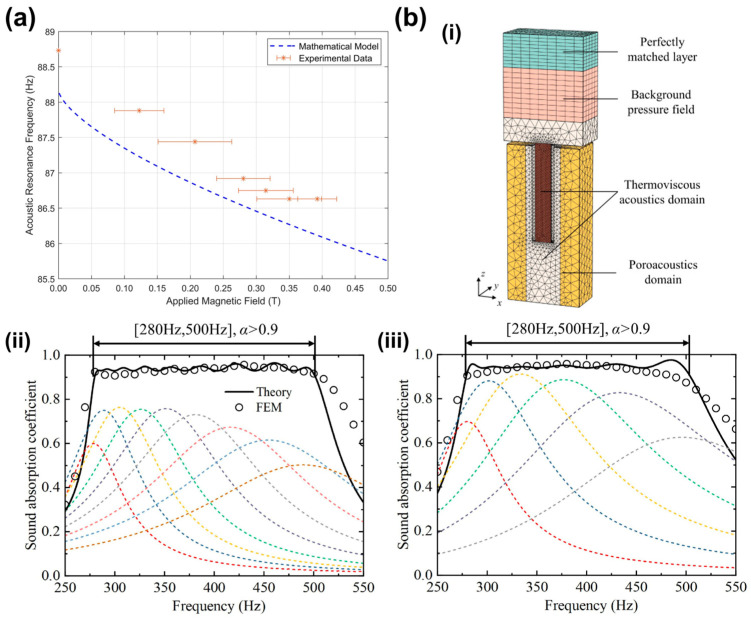
Magnetic field-induced property modulation and porous acoustic impedance tuning. (**a**) Acoustic resonance frequency as a function of applied magnetic field, for both experimental (red stars) and mathematical modeling approaches (dashed blue line). Adapted from Ref. [160] under CC BY 4.0 license. (https://doi.org/10.1038/s41598-024-65819-2). (**b**) Broadband low-frequency sound absorption via Helmholtz resonators with porous material lining. (**b**,**i**) Three-dimensional finite element model of the Helmholtz resonator with porous material lining (HRPL). Broadband absorption can be efficiently achieved by paralleling multiple HRPL subunits operating over different frequency ranges. (**b**,**ii**) Broadband sound absorption of sample 1, consisting of 9 subunits with porous material 1 lining. (**b**,**iii**) broadband sound absorption of sample 2, consisting of 6 subunits with porous material 3 lining. Adapted from Ref. [162] Copyright 2024, Elsevier. (https://doi.org/10.1016/j.jsv.2024.118330).

Gardiner et al. advanced this concept by utilizing 3D printing technology to create customized acoustic metamaterial units from a superparamagnetic resin (Figure 11a). By applying an external magnetic field, they physically altered the mechanical stiffness of the membrane. The experimental results showed a precise resonance frequency shift from 88.73 Hz to 86.63 Hz. This work is particularly significant for low-frequency acoustic control, offering a programmable route for developing adaptive acoustic metamaterials that can be tuned remotely without physical contact [160].

In 2025, Diksha Arora et al. fabricated SAW resonators based on magnetostrictive Ni-Mn-In and highly oriented piezoelectric PMN-PT layers [161]. By applying external magnetic and electric fields, the magnetostrictive and electrostrictive effects were respectively utilized to directly modulate the Young’s modulus of the materials, achieving resonance frequency tuning up to 10%. This dual-field approach enables independent control through magnetic and electric pathways, making the device suitable for reconfigurable RF devices and magnetic field sensing applications. The magnetostrictive component provides remote tunability without electrical contact, while the piezoelectric layer offers rapid electrical fine-tuning, combining the advantages of both modalities.

#### 4.2.3. Porous and Composite Acoustic Impedance Tuning

For broadband applications, frequency tuning can be achieved by regulating acoustic impedance and damping. Porous materials introduce viscous and thermal losses within their pore structures, and when integrated into resonators, they can modify the effective compliance and resistance of the system, thereby shifting the peak frequency and broadening the response bandwidth. Zhang et al. demonstrated this strategy by incorporating a porous lining into a Helmholtz resonator (Figure 11b). By adjusting the porosity and filling factor, they tuned both the absorption peak frequency and bandwidth, achieving efficient broadband sound absorption in the 280–500 Hz range [162]. This approach is valuable for medical noise-control environments and for improving the frequency selectivity of acoustic sensors.

### 4.3. Geometric Reconfiguration and Structural Design

Geometric reconfiguration is a highly versatile strategy for frequency tuning and often provides wider tuning ranges than electrical or material-based methods. Instead of modulating intrinsic material properties, this approach directly adjusts acoustic boundary conditions by changing structural parameters such as cavity height, rotation angle, perforation rate, or mass distribution. The resulting frequency shift can be described by the fundamental relations:

(3) $$f_{r}=\frac{c}{\lambda_{eff}}=\frac{c}{2L}\;(\mathrm{for}\;\mathrm{cavity}\;\mathrm{resonators})$$

(4) $$f_{r}=\frac{1}{2\pi }\sqrt{\frac{k_{eff}}{m_{eff}}}\;(\mathrm{for}\;\mathrm{mechanical}\;\mathrm{resonators})$$

where *L* is the characteristic dimension, $k_{eff}$ is the effective stiffness, and $m_{eff}$ is the effective mass. For medical applications, geometric tuning offers several advantages, including passive operation, immunity to electromagnetic interference, and broad scalability. However, these benefits are often accompanied by limitations in response speed and mechanical durability. This section focuses on recent advances in MEMS fabrication, soft materials, and bioinspired structures that enable adaptive acoustic sensing through geometric reconfiguration.

#### 4.3.1. Active Geometric Dimension Regulation

The most direct geometric tuning strategy is to mechanically adjust key structural dimensions, such as cavity height or channel width, thereby modifying acoustic impedance and resonance behavior.

Precision Cavity Control: The resonance frequency of acoustic cavities scales inversely with characteristic dimensions. Ni et al. demonstrated a topological acoustic metamaterial with tunable cavity heights in 2019. By compressing the structure, the cavity height was continuously adjusted from 12 mm to 4 mm, enabling dynamic switching between different topological acoustic states. This design allowed selective excitation of edge states and bulk modes, providing a new mechanism for acoustic signal routing and filtering in medical imaging applications requiring spatial selectivity [163].

Rotatable and Spiral Mechanisms: More agile geometric tuning strategies have also been developed. Chong et al. designed a reconfigurable rotation-symmetric unit based on Helmholtz resonance. By rotating internal and external cylinders to alter the structural geometry, they achieved continuous switching of operating frequencies and functions, including directional refraction, beam splitting, and focusing [164].

#### 4.3.2. Multi-Resonant Arrays and Biomimetic Designs

Instead of actively tuning a single resonator, biomimetic designs often employ arrays of resonators with spatially varied geometric parameters to achieve broadband passive coverage, analogous to the human cochlea.

**Figure 12:**
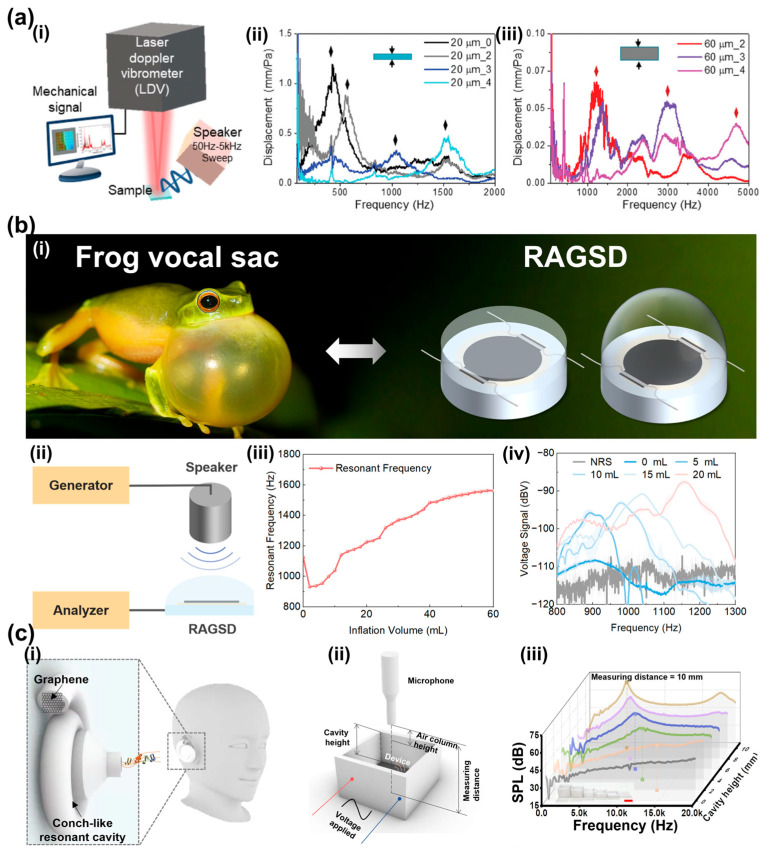
Multi-Resonant Arrays and Biomimetic Designs. (**a**) A Self-Powered Multiresonant Acoustic Sensing Array with Broad Bandwidth. (**a**,**i**) Schematic of the laser Doppler vibrometer (LDV) measurement setup for analyzing the frequency selectivity of the self-powered, miniaturized artificial basilar membrane (smBM). (**a**,**ii**) Generated voltage of 20 µm-thick poly-acrylonitrile (PAN) nanofiber (NF) mats combined with 0, 2, 3, and 4 spans. (**a**,**iii**) Displacement–frequency response spectra of 60 μm-thick polyacrylonitrile nanofiber membranes with 2, 3, and 4 spans. Adapted from Ref. [165], Copyright 2024 Wiley-VCH GmbH. (https://doi.org/10.1002/adfm.202306026). (**b**) Frog vocal sacs-inspired soft acoustic system with continuously tunable resonance. (**b**,**i**) Comparative illustration of vocal sac inflation in frogs and gas inflation in the RAGSD, used under license. (**b**,**ii**) The analysis system of RAGSD. (**b**,**iii**) The relationship between the resonant frequency of RAGSD and the inflation volume. (**b**,**iv**) Sound pressure level (SPL) response of RAGSD without resonant structure and with resonant structure inflated to 0/5/10/15/20 mL in the frequency domain, measured at a distance of 3 cm, with a standard speaker input voltage of 2 Vrms. Adapted from Ref. [166] under CC BY 4.0 license. (https://doi.org/10.1126/sciadv.adz5930). (**c**) Frequency-tunable sound amplification in a conch-like cavity. (**c**,**i**) Schematic diagram of wearing the C-cavity with a graphene device. (**c**,**ii**) Acoustic test platform of the Laser Scribed Graphene (LSG) device in a straight cavity. (**c**,**iii**) Sound emission performance of the LSG device in the different heights of cavities ranging from 0 to 10 mm (0/2/4/6/8/10 mm). Adapted from Ref. [167] under CC BY 4.0 license. (https://doi.org/10.1126/sciadv.adv2801).

Artificial Basilar Membranes: Lee et al. developed a self-powered multi-resonant artificial basilar membrane (ABM) with tunable inner boundary conditions (Figure 12a). Using micro-patterned elastic supports and a porous nanofiber membrane to regulate the resonance frequency of each channel, they achieved multi-channel tunability in the 400–3000 Hz range. This geometric gradient mimics the frequency resolution of the human ear and enables passive frequency selectivity without complex actuation circuitry [165].

Biomimetic Deformable Cavity Resonators: Inspired by the frequency-selective amplification mechanism of male frog vocal sacs, Liu et al. integrated LIG with a deformable elastic cavity to develop a resonance-adjustable graphene sound device (RAGSD) capable of continuous resonance tuning through cavity-volume modulation (Figure 12b). Unlike electrically biased tuning strategies, this device relies on structural tuning, achieving a broad tuning range of 91.18% from 922.12 to 1762.90 Hz [166]. The soft Ecoflex cavity provides a compliant exterior and conformal skin coupling, which is advantageous for wearable auscultation by improving acoustic transmission and reducing motion-induced degradation. Beyond integrating sound generation and stethoscopic sensing in a single flexible platform, the RAGSD selectively amplified weak high-frequency cardiac components. Supplementary results further showed that recognizable S1 and S2 heart sounds could still be retained during walking, suggesting that the combined effects of structural resonance tuning and soft skin coupling can suppress off-band disturbances and partially mitigate motion artifacts. To describe the nonlinear large deformation of the inflatable cavity, the authors established a Dynamic Continuously Tunable Electro-Mechano-Acoustical (DCT-EMA) equivalent-circuit model, which enabled predictive analysis of cavity-volume change, equivalent inductance/capacitance evolution, and resonance-frequency drift. Experimental validation showed a high prediction accuracy with an $R^{2}$ of 0.990, providing a useful design framework for soft tunable acoustic devices [166].

In flexible electronics, Wei et al. proposed a graphene-based wearable sound generator with a 3D-printed spiral cavity (Figure 12c). Although originally designed for sound generation, its resonator design also contributes to tunable acoustic sensing. By adjusting the spiral cavity height, the device achieved a wide tunable frequency range and enhanced low-frequency sound pressure, demonstrating effective acoustic amplification at both 1 kHz and 10 kHz [167].

#### 4.3.3. Mechanical Structural Deformation and Mass Regulation

**Figure 13:**
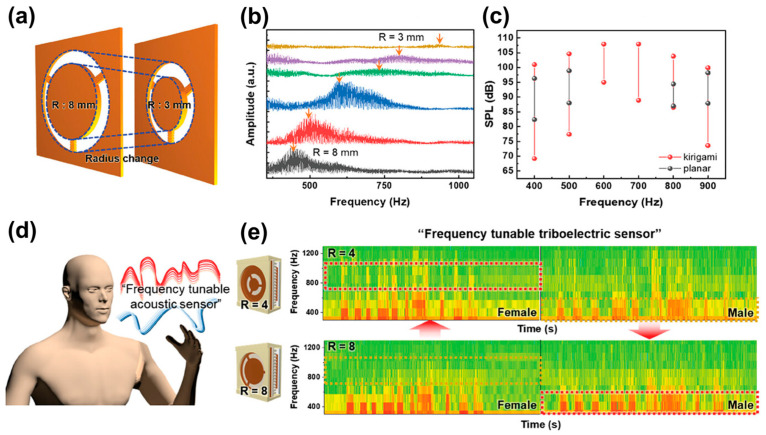
A self-powered, highly sensitive, and frequency-tunable triboelectric acoustic sensor. (**a**) Schematics of the frequency tuning system using the Kapton diaphragm by changing the radius of the mass-beam structure. (**b**) Fast-Fourier transforms results of the sensors after the frequency sweeping test on the Kapton diaphragms with different radii. (**c**) Dynamic range at the resonance frequency of each kirigami-structured mass-beam Kapton diaphragm with a different radius compared to the planar diaphragm. (**d**) Schematic of female and male voices applied to the fabricated sensors with Kapton diaphragms having different radii. (**e**) Short-time Fourier transform (STFT) data of fabricated sensors having different diaphragm radius (R = 4 mm and R = 8 mm) after applying male and female voice profiles. The red dotted boxes indicate the signal enhancement in the tuned resonance frequency regions. Adapted from Ref. [71] under CC BY 4.0 license. (https://doi.org/10.1002/adfm.202408344).

In addition to cavity-size modulation, tuning can also be achieved by directly reconfiguring the mass–stiffness distribution of the mechanical structure as illustrated in Figure 13. Inspired by the human cochlea, Kang et al. developed a biomimetic triboelectric acoustic sensor with adjustable mass and beam parameters, enabling controllable resonance-frequency tuning. This design mimics cochlear frequency selectivity and demonstrated the ability to distinguish voices at different frequencies, highlighting its potential for intelligent prosthetics and human–machine interaction (HMI) [71].

#### 4.3.4. Microstructural Modification and Variable Perforation

Frequency tuning can also be achieved by modifying microstructural parameters that affect acoustic impedance. Cheng et al. developed a hexagonal multi-cavity acoustic metamaterial with adjustable perforation rates, enabling tunable resonance points and low-frequency noise control [168].

### 4.4. Summary: Toward Intelligent and Adaptive Acoustic Sensing

The transition from fixed-frequency resonators to tunable acoustic architectures represents an important advance in medical sensing. As discussed above, resonance frequency can be modulated through three main pathways: electrical tuning, material/property-based tuning, and geometric reconfiguration, each with distinct functional advantages for addressing the spectral diversity of physiological acoustic signals.

#### 4.4.1. Synthesis of Tuning Mechanisms

State-of-the-art tuning strategies fall into three principal domains based on their underlying physics (see Table 3):

Because the tunable acoustic sensors discussed in this review differ substantially in operating frequency range, transduction mechanism, and target application, a strict one-to-one comparison based only on absolute performance metrics is not sufficiently informative. To enable a more consistent comparison, the key performance parameters are reorganized in a unified format in Table 3. Specifically, the tuning range is expressed, wherever possible, as the relative resonance shift $(\Delta f/f_{0})\times 100\%$; the reported *Q* value is retained for quality-factor comparison; tuning sensitivity and sensing sensitivity are listed separately to distinguish their different physical meanings; and response time is included only when explicitly reported in the original studies, otherwise marked as “NR”. In addition, a dedicated column for the tuning method is included to indicate the physical mechanism of resonance reconfiguration.

Within this framework, different tuning strategies can be compared not only by numerical performance, but also by their distinct functional characteristics. Electrical tuning is generally associated with high integrability, programmability, and real-time controllability, making it particularly suitable for on-chip and reconfigurable sensing systems [148]. Material/property-based tuning is attractive for adaptive and multifunctional platforms because the acoustic response can be modulated through changes in intrinsic material properties or externally coupled fields [160]. By contrast, structural/geometric tuning often provides larger tuning ranges without continuous external bias and is inherently compatible with soft and flexible device architectures [71,166]. This feature is especially relevant to wearable physiological acoustic monitoring, where conformal skin coupling can improve signal quality and operational stability. Therefore, the purpose of the table is not only to compare numerical parameters, but also to clarify the functional merits and translational potential of different tuning strategies.

Electrical tuning provides a direct and precise way to modulate resonance frequency, enabling reversible and controllable spectral adjustment. For example, Pang et al. reported that an FBAR achieved 1.47% tuning at 1.5 GHz under a 7 V DC bias [148], while Nastro et al. showed that a piezoelectric MEMS acoustic transducer produced a resonance shift of about 70 Hz near 5.5 kHz under a DC bias from −4 to +4 V [151]. These results demonstrate the strong frequency-modulation capability of electrically tuned devices. However, their application in physiological monitoring, especially in wearable or implantable systems, remains limited at present, mainly because external biasing introduces additional power consumption, thermal concerns, and system complexity. Nevertheless, their precise and reversible tuning capability still makes them promising for future physiological signal monitoring requiring adaptive spectral matching.

Material-Based Tuning exploits intrinsic material responses to external stimuli. Chemical infiltration through mesoporous structures achieves reversible tuning up to 60% via humidity-responsive mechanisms, while magnetostrictive materials provide non-contact magnetic field modulation (2–3 Hz frequency shifts). These passive mechanisms excel in breath analysis for volatile organic compound detection, post-surgical calibration of enclosed implants, and self-powered monitoring, where electrical infrastructure is limited [159,160,162].

Geometric Reconfiguration through physical modification of structural dimensions (cavity heights, rotation angles, perforation rates, and mass-beam distributions) offers the widest tuning ranges, spanning multiple octaves [165,167]. Precision cavity control enables topological acoustic state manipulation; rotatable Helmholtz resonators achieve multi-functional beam steering; biomimetic mass-beam structures provide cochlear-inspired frequency selectivity. Applications include wearable health monitors, multi-channel artificial basilar membranes (400–3000 Hz coverage), intelligent prosthetics with Human–Machine Interaction, and broadband low-frequency noise control (280–500 Hz).

#### 4.4.2. Performance Implications of Tunability in Physiological Acoustic Sensing

More importantly, the performance advantage of FTAS should be understood as a trade-off between absolute peak sensitivity and spectral selectivity. For a high-*Q* resonant sensor, the bandwidth is approximately governed by

(5) $$Q\approx \frac{f_{r}}{\Delta f_{3dB}}$$

which indicates that resonance tuning shifts a narrow passband toward the target frequency region. In practice, enlarging the tuning range often makes it difficult to maintain the same peak response at all tuned states, so the absolute sensitivity may decrease under some conditions [169,170]. Nevertheless, this movable narrowband response can still selectively enhance target frequency components while suppressing off-band interference.

This feature is particularly relevant to physiological acoustic sensing, where weak heart and respiratory sounds are often masked by broadband noise, unstable body coupling, and motion artifacts. Compared with untunable or broadband sensors, FTAS can shift its sensitive passband toward the spectral region of interest, thereby enhancing in-band physiological signals and relatively suppressing off-band noise. However, this advantage is conditional rather than universal, because its effectiveness depends on the spectral separation between target signals and artifacts, as well as on the resonance bandwidth and tuning stability of the device.

Resonance-frequency tunability is also fundamentally different from conventional software filtering. Digital filtering mainly removes unwanted spectral components after signal acquisition, whereas resonance tuning acts at the hardware level by shifting the sensor response peak toward the desired band. In this way, FTAS can provide pre-digitization frequency-selective enhancement and may improve the capture of weak pathological acoustic components from noisy backgrounds.

At the system level, tunability should not be regarded as incompatible with broadband acquisition. In some FTAS, the sensing element provides broadband signal transduction, while the tuning component introduces resonance reconfiguration for selective enhancement. A representative example is the frog-vocal-sac-inspired RAGSD, in which LIG is integrated with a deformable cavity to achieve continuous resonance tuning from 922.12 to 1762.90 Hz [166]. In this design, the LIG element supports broadband acoustic transduction, whereas the flexible cavity provides adjustable spectral selectivity, allowing both functions to be integrated in a single platform. The study further showed that resonance matching could amplify weak high-frequency heart-sound components during stethoscopic sensing.

At the same time, effective suppression of noise and motion artifacts does not rely on tunability alone. Flexible, skin-conformal device designs can improve mechanical coupling and reduce contact instability, while signal-processing methods such as denoising and machine learning can further suppress residual interference. Therefore, the practical value of FTAS in physiological monitoring should be understood as the combined effect of resonance tuning, improved body–sensor interfacing, and signal-processing support. As medical applications of these sensors are still at an early stage, their significance currently lies more in their translational potential than in established clinical utility.

#### 4.4.3. The Potential Applications of FTAS in Acoustic Signal Monitoring

Cardiopulmonary monitoring is currently the most clinically relevant application direction for tunable acoustic platforms. Because heart and lung sounds occupy partially overlapping frequency bands and are easily corrupted by environmental noise, motion artifacts, and variable skin contact, fixed-band acoustic sensors often struggle to provide stable and selective signal acquisition. Tunable resonant devices offer a promising alternative by enabling adaptive spectral matching to target physiological sounds. For example, a multiresonant artificial basilar membrane with tunable inner boundary conditions achieved selectable bands from 400 to 3000 Hz, illustrating the potential of multichannel frequency discrimination in a compact architecture [165]. A frog-vocal-sac-inspired soft acoustic system further demonstrated continuous resonance tuning from 922.12 to 1762.90 Hz and wearable stethoscopic sensing with amplification of weak high-frequency cardiac components; when integrated with a deep-learning classifier, it achieved 99.375% accuracy across four clinical classes [166]. Electrically tunable piezoelectric MEMS transducers also support the feasibility of adaptive cardiopulmonary acoustic monitoring [151]. Overall, the main value of tunable acoustic platforms in this area lies in adaptive spectral enhancement, although their clinical translation still depends on improved robustness against environmental interference, motion artifacts, and body-interface instability.

In addition, Kang et al. developed a cochlea-inspired frequency-tunable triboelectric acoustic sensor in which the resonance frequency is adjusted by changing the radius of a kirigami-structured mass–beam Kapton diaphragm, enabling frequency-selective discrimination of male and female voice components. This mechanically tunable, cochlea-mimetic strategy suggests a possible route toward customized respiratory acoustic sensing, where the resonance characteristics could be tailored to different subjects or to sex-related spectral differences in physiological sounds. Although biomedical implementation has not yet been demonstrated, its reconfigurable structural selectivity indicates promise for future personalized physiological acoustic monitoring platforms [71].

## 5. Conclusions

### 5.1. Comparative Advantages: From Passive Reception to Active Interrogation

The tunable resonant acoustic sensors reviewed above show strong potential for adapting to diverse physiological signals through dynamic frequency reconfiguration. However, several technical challenges still need to be addressed before broad clinical translation can be achieved [171].

Compared with current fixed-frequency sensors, tunable resonant devices provide a distinct operational strategy. Traditional broadband transducers generally capture a wide range of frequency components with nearly uniform sensitivity [4], which is advantageous when pathological spectral features are uncertain. In contrast, tunable sensors introduce hardware-level frequency selectivity, enabling targeted enhancement of specific bands and suppression of off-band interference before signal digitization [172]. This advantage is mainly reflected in two aspects.

#### 5.1.1. Adaptive Signal Enhancement

By aligning resonance frequency with pathological features identified in Section 2, cardiac murmurs (200–2000 Hz), respiratory crackles (>650 Hz), or voice disorders (altered formants at 2 to 5 kHz), tunable sensors achieve selective amplification at the band of interest [70]. This hardware pre-filtering fundamentally differs from digital post-processing: it reduces dynamic range requirements for analog-to-digital converters, lowers power consumption (12-bit ADCs suffice vs. 16–24 bit for broadband capture), and improves signal-to-noise ratio before quantization noise corrupts weak signals [173].

#### 5.1.2. Noise Rejection Through Spectral Discrimination

In clinical environments contaminated by ambient noise, tunable sensors actively suppress out-of-band components through physical resonance mechanisms rather than relying solely on computational filtering [174]. The RAGSD platform’s 91.18% tuning range (922.12–1762.90 Hz) enables precise isolation of target signals from overlapping noise sources [166].

### 5.2. Fundamental Challenges and Technical Barriers

Frequency tunability requires additional functional layers and actuation mechanisms that are absent in passive sensors, which inevitably increases device complexity. For example, electrical tuning often depends on voltage-control circuits, dielectric layers, and precisely patterned electrodes, whereas geometric reconfiguration requires deformable membranes, inflation channels, or rotational actuators, all of which introduce extra fabrication steps and potential failure modes [142].

The achievable tuning range is also constrained by intrinsic material properties and structural design. Electrical tuning based on capacitance modulation typically provides only limited relative frequency shifts, which may be insufficient for covering broad physiological acoustic bands [175]. Material-based approaches often suffer from relatively slow response, making real-time clinical monitoring more difficult. Geometric methods can achieve much larger tuning ranges, but they also raise concerns about long-term mechanical fatigue [175,176].

In addition, different tuning strategies show different sensitivities to realistic physiological disturbances. Electrically tuned resonant devices can be affected by bias instability and temperature-dependent resonance drift [177]. Material-based or triboelectric-related tuning is more vulnerable to humidity and perspiration, which may reduce charge retention and output stability. Geometrically reconfigurable and flexible structural designs are more strongly influenced by motion-induced boundary changes, skin coupling, and tissue loading, although conformal soft structures may help alleviate some of these effects. For example, the RAGSD developed by Liu et al. achieved continuous tuning from 922.12 to 1762.90 Hz and demonstrated the potential of flexible structural tuning for pathological heart-sound monitoring [166].

### 5.3. Future Directions: Enabling Technologies and Strategic Pathways

Building upon the existing body of review articles and original research studies, the present review systematically summarizes the development of acoustic sensors from conventional architectures to resonance-frequency-tunable systems. More importantly, it further highlights the application potential of resonance-frequency-tunable acoustic sensors and discusses their future development directions in physiological acoustic monitoring, which represents a key novelty of this review.

Addressing the aforementioned challenges requires strategic integration of emerging materials and advanced fabrication techniques.

Three key development pathways emerge:

(1) Advanced Materials for Enhanced Tunability

Novel material systems promise to overcome current tuning range and stability limitations. Two-dimensional materials (graphene, MoS_2_) enable voltage-controlled mechanical property modulation through electrostatic gating, achieving >20% frequency shifts with microsecond response while maintaining atomic-scale thickness for flexible integration.

(2) Biomimetic Architectures for Adaptive Sensing

Biologically inspired designs will enhance functionality and adaptability. Biomimetic resonance structures leveraging natural frequency-tunable mechanisms, such as frog vocal sacs (dynamic cavity volume modulation for mating call frequency control) and spiral cochlear geometries (gradient impedance matching across frequency bands provide design blueprints for adaptive acoustic sensors. These biological archetypes demonstrate how structural reconfiguration (membrane tension variation, chamber geometry adjustment) achieves wide-range frequency tuning while maintaining mechanical robustness, guiding the development of deformable resonator architectures that mimic nature’s solutions to adaptive sound processing.

(3) Patient-Specific Customizable Platforms

Personalized frequency-tunable sensors represent a promising evolution toward precision medicine, because they may help address the inter-patient variability that often limits fixed-configuration devices. Anatomical differences, such as body habitus, chest-wall thickness, skin impedance, and tissue-specific attenuation, can substantially alter the acoustic coupling between physiological sound sources and the sensor [178], while demographic and pathological factors may further modify the spectral characteristics of target signals. In principle, customizable tunable platforms could address these challenges through adaptive calibration protocols, for example, by using impedance-related coupling information to guide resonance selection and by adjusting the resonance frequency according to the spectral characteristics of the incoming physiological signal. In this context, the value of frequency-tunable devices lies not only in resonance adjustment itself, but also in their potential front-end signal-conditioning capability.

It should also be noted that many currently reported tunable resonant sensors were originally developed as general acoustic or bioinspired devices, and their operating bands do not always directly match the main frequency ranges of physiological acoustic signals. Therefore, their present significance lies more in providing tunable sensing strategies than in representing clinically mature physiological monitoring systems. Nevertheless, recently reported bioinspired tunable MEMS devices have demonstrated dynamic resonance tuning together with hardware-based frequency analysis and adaptive bandpass characteristics, suggesting that tunable resonant hardware may support front-end frequency-selective enhancement while reducing dependence on multi-sensor filter-bank architectures [179]. In this regard, combining tunable hardware with Artificial Intelligence (AI) is especially promising: the hardware can enhance target-band signals and suppress off-band noise, while AI can further support adaptive optimization for different users and conditions. The RAGSD reported by Liu et al. [166], which amplified weak high-frequency cardiac sounds and was integrated with deep learning for intelligent auscultation, provides an early example of this direction.

More broadly, tunable acoustic sensors may offer important advantages for multimodal physiological monitoring by enabling adaptive spectral targeting. A single device with adjustable resonance could potentially support cardiac, respiratory, and gastrointestinal monitoring by sequentially matching different physiological frequency bands, thereby reducing hardware complexity and improving the practicality of wearable continuous monitoring systems.

## Figures and Tables

**Table 1:** Sensor categories and corresponding sections covered in this review.

Sensor Group	Sensor Class	Section
Fixed-frequency acoustic sensors	Piezoelectric acoustic sensors	Section 3.1
	Capacitive MEMS acoustic sensors	Section 3.2
	Piezoresistive acoustic sensors	Section 3.3
	Triboelectric acoustic sensors	Section 3.4
FTAS	Electrical tunable acoustic sensors	Section 4.1
	Material and property-tunable acoustic sensors	Section 4.2
	Geometrically reconfigurable acoustic sensors	Section 4.3

**Table 2:** Summary of the frequency ranges of four representative physiological acoustic signals.

Physiological Acoustic Signal	Normal Range	Abnormal Range	Overall Range
HSs	S1 and S2 mainly: 20–150 Hz	Clicks: 100–300 Hz; Murmurs: 300–1200 Hz, highly at 2000 Hz	20–2000 Hz
BrSs	Normal breath sounds: 100–500 Hz	Wheezes and stridor: 400 Hz to 2500 Hz	100–2500 Hz
BoSs	Most bowel sounds: 50–300 Hz	Pathological or fluid-related components: 300–1000 Hz	50–1000 Hz
VSs	Fundamental frequency 80–180 Hz (male); 160–300 Hz (female)	Harmonics and pathological noise components: 200–4000 Hz	80–1200 Hz (male); 160–2000 Hz (female)

**Table 3:** Comparison and summary of the three tuning mechanisms. “Not Reported (NR)” indicates that the parameter was not mentioned in the paper.

Tuning Strategy	Representative Platform	Tuning Method	Tuning Range ((∆ff0) × 100%) or Reported Shift	Q Factor/ Q-Retention	Tuning Sensitivity	Sensing Sensitivity	Reaction Time	Reference
Electrical tuning	FBAR integrated with an electrostatic MEMS actuator	Electrostatic actuation	22.5 MHz at 1.5 GHz (1.47%)	160–304	about 8 ppm/V at 3.4 GHz	NR	NR	[148]
	Piezoelectric MEMS acoustic transducer	DC-bias-induced piezoelectric stress tuning	tunable within $V_{b}=\pm 8\;V$ (A frequency shift of ±70 Hz)	NR	8.7 ± 0.5 Hz/V (transmission); 7.8 ± 0.9 Hz/V (reception)	NR	NR	[151]
	LiNbO_3_ resonator	DC-bias-induced nonlinear piezoelectric tuning	about 0.4%	NR	6–12 kHz/V reported	NR	NR	[74]
Material/property-based tuning	Magnetic membrane acoustic metamaterial	Magnetic-field-controlled stiffness tuning	88.73 → 86.63 Hz (~2.37%)	NR	NR	NR	NR	[160]
Geometric tuning	Multi-resonant artificial basilar membrane	Inner boundary condition tuning	400–3000 Hz selectable bands	NR	NR	frequency selectivity reported	NR	[165]
	Frog-vocal-sac-inspired soft acoustic system	Internal cavity-volume reconfiguration	922.12–1762.90 Hz (91.18%)	NR	NR	25.34 dB SPL gain at resonance	NR	[166]

## Data Availability

No new original research data were created or analyzed in this review article. Therefore, data sharing is not applicable to this study. The review is based on previously published literature, and the relevant data supporting the discussed findings can be found in the original cited references.

## References

[1] Leng S., Tan R.S., Chai K.T., Wang C., Ghista D., Zhong L. (2015). The Electronic Stethoscope. Biomed. Eng. Online.

[2] Longfu Z., Yi S., Sun H., Zheng L., Dapeng H., Yonghe H. Identification of Bowel Sound Signal with Spectral Entropy Method. Proceedings of the 2015 12th IEEE International Conference on Electronic Measurement & Instruments (ICEMI).

[3] Yoo J.Y., Oh S., Shalish W., Maeng W.Y., Cerier E., Jeanne E., Chung M.K., Lv S., Wu Y., Yoo S. (2023). Wireless Broadband Acousto-Mechanical Sensing System for Continuous Physiological Monitoring. Nat. Med..

[4] Mallegni N., Molinari G., Ricci C., Lazzeri A., La Rosa D., Crivello A., Milazzo M. (2022). Sensing Devices for Detecting and Processing Acoustic Signals in Healthcare. Biosensors.

[5] Liu T., Mao Y., Dou H., Zhang W., Yang J., Wu P., Li D., Mu X. (2025). Emerging Wearable Acoustic Sensing Technologies. Adv. Sci..

[6] Lin Z., Duan S., Liu M., Dang C., Qian S., Zhang L., Wang H., Yan W., Zhu M. (2024). Insights into Materials, Physics, and Applications in Flexible and Wearable Acoustic Sensing Technology. Adv. Mater..

[7] Xue J., Rong J., Guo W., Xue K., Xing E., Wen H., Zhou Y., Liu W., Tang J., Liu J. (2025). High-Sensitivity Acoustic Sensing Based on Mechanical Resonance Modulation of Whispering Gallery Mode Optical Resonator. Opt. Eng..

[8] Yaseen S.G.-Y., Kwon S. (2018). Classification of Heart Sound Signal Using Multiple Features. Appl. Sci..

[9] Yoshino H., Abe Y., Yoshino T., Ohsato K. (1990). Clinical Application of Spectral Analysis of Bowel Sounds in Intestinal Obstruction. Dis. Colon Rectum.

[10] Ching S.S., Tan Y.K. (2012). Spectral Analysis of Bowel Sounds in Intestinal Obstruction Using an Electronic Stethoscope. World J. Gastroenterol..

[11] Emmanouilidou D., Patil K., West J., Elhilali M. A Multiresolution Analysis for Detection of Abnormal Lung Sounds. Proceedings of the 2012 Annual International Conference of the IEEE Engineering in Medicine and Biology Society.

[12] Cabot R.C., Dodge H.F. (1925). Frequency Characteristics of Heart and Lung Sounds. J. Am. Med. Assoc..

[13] Murry T., Singh S. (1980). Multidimensional Analysis of Male and Female Voices. J. Acoust. Soc. Am..

[14] Latinus M., Taylor M.J. (2012). Discriminating Male and Female Voices: Differentiating Pitch and Gender. Brain Topogr..

[15] Titze I.R., Mapes S., Story B. (1994). Acoustics of the Tenor High Voice. J. Acoust. Soc. Am..

[16] Garnier M., Henrich N., Smith J., Wolfe J. (2010). Vocal Tract Adjustments in the High Soprano Range. J. Acoust. Soc. Am..

[17] Hanna I.R., Silverman M.E. (2002). A History of Cardiac Auscultation and Some of Its Contributors. Am. J. Cardiol..

[18] Rennoll V., McLane I., Emmanouilidou D., West J., Elhilali M. (2020). Electronic Stethoscope Filtering Mimics the Perceived Sound Characteristics of Acoustic Stethoscope. IEEE J. Biomed. Health Inform..

[19] McLane I., Emmanouilidou D., West J.E., Elhilali M. (2021). Design and Comparative Performance of a Robust Lung Auscultation System for Noisy Clinical Settings. IEEE J. Biomed. Health Inform..

[20] Yilmaz G., Rapin M., Pessoa D., Rocha B.M., de Sousa A.M., Rusconi R., Carvalho P., Wacker J., Paiva R.P., Chetelat O. (2020). A Wearable Stethoscope for Long-Term Ambulatory Respiratory Health Monitoring. Sensors.

[21] Lee S.H., Kim Y.S., Yeo W.H. (2021). Advances in Microsensors and Wearable Bioelectronics for Digital Stethoscopes in Health Monitoring and Disease Diagnosis. Adv. Healthc. Mater..

[22] Wu Y.C., Han C.C., Chang C.S., Chang F.L., Chen S.F., Shieh T.Y., Chen H.M., Lin J.Y. (2022). Development of an Electronic Stethoscope and a Classification Algorithm for Cardiopulmonary Sounds. Sensors.

[23] Hu Y., Kim E.G., Cao G., Liu S., Xu Y. (2014). Physiological Acoustic Sensing Based on Accelerometers: A Survey for Mobile Healthcare. Ann. Biomed. Eng..

[24] Rao A., Huynh E., Royston T.J., Kornblith A., Roy S. (2019). Acoustic Methods for Pulmonary Diagnosis. IEEE Rev. Biomed. Eng..

[25] Gupta P., Moghimi M.J., Jeong Y., Gupta D., Inan O.T., Ayazi F. (2020). Precision Wearable Accelerometer Contact Microphones for Longitudinal Monitoring of Mechano-Acoustic Cardiopulmonary Signals. npj Digit. Med..

[26] Kirchner J., Souilem S., Fischer G. Wearable System for Measurement of Thoracic Sounds with a Microphone Array. Proceedings of the 2017 IEEE SENSORS.

[27] Tamborini A., Gharib M. (2024). Listening to Heart Sounds through the Pressure Waveform. Sci. Rep..

[28] Oktivasari P., Haryanto F. (2022). A Multimodal Model of Ecg and Heart Sound Signal by Considering Normal and Abnormal Heart. JOIV Int. J. Inform. Vis..

[29] Piemme T.E., Barnett G.O., Dexter L. (1966). Relationship of Heart Sounds to Acceleration of Blood Flow. Circ. Res..

[30] Altuve M., Suárez L., Ardila J. (2020). Fundamental Heart Sounds Analysis Using Improved Complete Ensemble Emd with Adaptive Noise. Biocybern. Biomed. Eng..

[31] Arnott P., Pfeiffer G., Tavel M. (1984). Spectral Analysis of Heart Sounds: Relationships between Some Physical Characteristics and Frequency Spectra of First and Second Heart Sounds in Normals and Hypertensives. J. Biomed. Eng..

[32] Cherif L.H., Debbal S. (2020). Spectro-Temporal Characteristics of the Internal Components Mitral (M1) and Tricuspid (T1) of the First Heart Sound (S1) Pulmonary (P2) and Aortic (A2) of the Second Cardiac Sound (S2) Using the Continuous Wavelet Transform (Cwt). Ann. Cardiol. Vasc. Med..

[33] Nygaard H., Thuesen L., Hasenkam J.M., Pedersen E.M., Paulsen P.K. (1993). Assessing the Severity of Aortic Valve Stenosis by Spectral Analysis of Cardiac Murmurs (Spectral Vibrocardiography). Part I: Technical Aspects. J. Heart Valve Dis..

[34] Zhu C., Seo J.-H., Mittal R. (2018). Computational Modelling and Analysis of Haemodynamics in a Simple Model of Aortic Stenosis. J. Fluid. Mech..

[35] Wang Z., Seo J.H., Mittal R. (2022). Mitral Valve Regurgitation Murmurs—Insights from Hemoacoustic Computational Modeling. Fluids.

[36] Nemani L., Pechetty R. (2020). Additional Heart Sounds–Part 2 (Clicks, Opening Snap and More). Indian J. Cardiovasc. Dis. Women.

[37] Aboulhosn J., Child J.S. (2006). Left Ventricular Outflow Obstruction: Subaortic Stenosis, Bicuspid Aortic Valve, Supravalvar Aortic Stenosis, and Coarctation of the Aorta. Circulation.

[38] Pramono R.X.A., Bowyer S., Rodriguez-Villegas E. (2017). Automatic Adventitious Respiratory Sound Analysis: A Systematic Review. PLoS ONE.

[39] Gavriely N., Palti Y., Alroy G. (1981). Spectral Characteristics of Normal Breath Sounds. J. Appl. Physiol. Respir. Environ. Exerc. Physiol..

[40] Gupta P., Wen H., Di Francesco L., Ayazi F. (2021). Detection of Pathological Mechano-Acoustic Signatures Using Precision Accelerometer Contact Microphones in Patients with Pulmonary Disorders. Sci. Rep..

[41] Oliveira A., Marques A. (2014). Respiratory Sounds in Healthy People: A Systematic Review. Respir. Med..

[42] Ohshimo S., Sadamori T., Tanigawa K. (2016). Innovation in Analysis of Respiratory Sounds. Ann. Intern. Med..

[43] Park J.S., Kim K., Kim J.H., Choi Y.J., Kim K., Suh D.I. (2023). A Machine Learning Approach to the Development and Prospective Evaluation of a Pediatric Lung Sound Classification Model. Sci. Rep..

[44] Gavriely N., Palti Y., Alroy G., Grotberg J.B. (1984). Measurement and Theory of Wheezing Breath Sounds. J. Appl. Physiol. Respir. Environ. Exerc. Physiol..

[45] Kim Y., Hyon Y., Jung S.S., Lee S., Yoo G., Chung C., Ha T. (2021). Respiratory Sound Classification for Crackles, Wheezes, and Rhonchi in the Clinical Field Using Deep Learning. Sci. Rep..

[46] Cengizler C. (2025). A Novel Spectral Analysis-Based Grading System for Gastrointestinal Activity. PLoS ONE.

[47] Deng X., Xu Y., Zou Y. (2025). Numerical Modeling of Bowel Sound Propagation: Impact of Abdominal Tissue Properties. Appl. Sci..

[48] Redij R., Kaur A., Muddaloor P., Sethi A.K., Aedma K., Rajagopal A., Gopalakrishnan K., Yadav A., Damani D.N., Chedid V.G. (2023). Practicing Digital Gastroenterology through Phonoenterography Leveraging Artificial Intelligence: Future Perspectives Using Microwave Systems. Sensors.

[49] Haraguchi T., Emoto T., Hirayama T., Imai Y., Kato M., Hirano T. (2023). Peak-Frequency Histogram Similarity of Bowel Sounds for the Evaluation of Intestinal Conditions. Appl. Sci..

[50] Wang Z., Zhou W., Shu T., Xue Q., Zhang R., Wiercigroch M. (2022). Modelling of Low-Frequency Acoustic Wave Propagation in Dilute Gas-Bubbly Liquids. Int. J. Mech. Sci..

[51] Zhang Z. (2016). Cause-Effect Relationship between Vocal Fold Physiology and Voice Production in a Three-Dimensional Phonation Model. J. Acoust. Soc. Am..

[52] Stevens K.N. (2000). Acoustic Phonetics.

[53] Yamauchi A., Yokonishi H., Imagawa H., Sakakibara K., Nito T., Tayama N., Yamasoba T. (2015). Quantitative Analysis of Digital Videokymography: A Preliminary Study on Age- and Gender-Related Difference of Vocal Fold Vibration in Normal Speakers. J. Voice.

[54] Shrivas A., Deshpande S., Gidaye G., Nirmal J., Ezzine K., Frikha M., Desai K., Shinde S., Oza A.D., Burduhos-Nergis D.D. (2022). Employing Energy and Statistical Features for Automatic Diagnosis of Voice Disorders. Diagnostics.

[55] Liu B., Lei J., Wischhoff O.P., Smereka K.A., Jiang J.J. (2025). Acoustic Character Governing Variation in Normal, Benign, and Malignant Voices. Folia Phoniatr. Logop..

[56] Choudry M., Stead T.S., Mangal R.K., Ganti L. (2022). The History and Evolution of the Stethoscope. Cureus.

[57] Roguin A. (2006). Rene Theophile Hyacinthe Laënnec (1781–1826): The Man Behind the Stethoscope. Clin. Med. Res..

[58] Corwin A. (1895). Corwin’s Double Binaural Stethoscope. J. Am. Med. Assoc..

[59] McKusick V.A., Sharpe W.D., Warner A.O. (1957). An Exhibition of the History of Cardiovascular Sound Including the Evolution of the Stethoscope. Bull. Hist. Med..

[60] Littmann D. (1961). An Approach to the Ideal Stethoscope. JAMA.

[61] Mills G.A., Nketia T.A., Oppong I.A., Kaufmann E.E. (2012). Wireless Digital Stethoscope Using Bluetooth Technology. Int. J. Eng. Sci. Technol..

[62] Lee S.H., Kim Y.S., Yeo M.K., Mahmood M., Zavanelli N., Chung C., Heo J.Y., Kim Y., Jung S.S., Yeo W.H. (2022). Fully Portable Continuous Real-Time Auscultation with a Soft Wearable Stethoscope Designed for Automated Disease Diagnosis. Sci. Adv..

[63] Joyashiki T., Wada C. (2020). Validation of a Body-Conducted Sound Sensor for Respiratory Sound Monitoring and a Comparison with Several. Sensors.

[64] Roh K.M., Awosika A., Millis R.M. (2024). Advances in Wearable Stethoscope Technology: Opportunities for the Early Detection and Prevention of Cardiovascular Diseases. Cureus.

[65] Zauli M., Peppi L.M., Di Bonaventura L., Arcobelli V.A., Spadotto A., Diemberger I., Coppola V., Mellone S., De Marchi L. (2023). Exploring Microphone Technologies for Digital Auscultation Devices. Micromachines.

[66] Lee H.K., Park S.U., Kong S., Ryu H., Kim H.B., Lee S.H., Kang D., Shin S.H., Yu K.J., Cho J. (2024). Real-Time Deep Learning-Assisted Mechano-Acoustic System for Respiratory Diagnosis and Multifunctional Classification. NPJ Flex. Electron..

[67] Qiu S., Xiao T., Li Y., Yu X., Wu S., Zhang Y., Lin Y., Zhao N. (2025). A Multi-Modal Smart Chest Patch for Real-Time Cardiopulmonary Monitoring and Anomaly Detection. Sci. China Mater..

[68] Ficek J., Radzikowski K., Nowak J.K., Yoshie O., Walkowiak J., Nowak R. (2021). Analysis of Gastrointestinal Acoustic Activity Using Deep Neural Networks. Sensors.

[69] Griffin L., Luque J.S., Szynkiewicz S.H., Kamarunas E. (2025). Acoustic Measures of Voice Perturbation Offer Limited Value as Standalone Indicators of Laryngeal Penetration or Aspiration. Arch. Phys. Med. Rehabil..

[70] Han J.H., Kwak J.-H., Joe D.J., Hong S.K., Wang H.S., Park J.H., Hur S., Lee K.J. (2018). Basilar Membrane-Inspired Self-Powered Acoustic Sensor Enabled by Highly Sensitive Multi Tunable Frequency Band. Nano Energy.

[71] Kang D.h., Lee H., Song M., Ro Y.G., Kwak M.S., Kim J., Jung G., Park J., Kim Y.R., Lee J. (2024). A Self-Powered, Highly Sensitive, and Frequency-Tunable Triboelectric Acoustic Sensor Inspired by the Human Cochlea. Adv. Funct. Mater..

[72] Tian Z., Shen C., Li J., Reit E., Bachman H., Socolar J.E.S., Cummer S.A., Jun Huang T. (2020). Dispersion Tuning and Route Reconfiguration of Acoustic Waves in Valley Topological Phononic Crystals. Nat. Commun..

[73] Körner J. (2018). Effective Sensor Properties and Sensitivity Considerations of a Dynamic Co-Resonantly Coupled Cantilever Sensor. Beilstein J. Nanotechnol..

[74] Shao L., Zhu D., Colangelo M., Lee D., Sinclair N., Hu Y., Rakich P.T., Lai K., Berggren K.K., Lončar M. (2022). Electrical Control of Surface Acoustic Waves. Nat. Electron..

[75] Ozevin D. (2020). Mems Acoustic Emission Sensors. Appl. Sci..

[76] Chen Y., Zhang X., Lu C. (2024). Flexible Piezoelectric Materials and Strain Sensors for Wearable Electronics and Artificial Intelligence Applications. Chem. Sci..

[77] Srinivasaraghavan Govindarajan R., Rojas-Nastrucci E., Kim D. (2021). Surface Acoustic Wave-Based Flexible Piezocomposite Strain Sensor. Crystals.

[78] Wang Q., Zhang Y., Cheng S., Wang X., Wu S., Liu X. (2024). Mems Acoustic Sensors: Charting the Path from Research to Real-World Applications. Micromachines.

[79] Wu Y., Tang C.Y., Wang S., Guo J., Jing Q., Liu J., Ke K., Wang Y., Yang W. (2025). Biomimetic Heteromodulus All-Fluoropolymer Piezoelectric Nanofiber Mats for Highly Sensitive Acoustic Detection. ACS Appl. Mater. Interfaces.

[80] Sawane M., Prasad M. (2025). Design and Optimization of Piezoelectric Diaphragm for Self-Powered Acoustic Sensor. Eng. Res. Express.

[81] Campo-Valera M., Asorey-Cacheda R., Rodriguez-Rodriguez I., Villo-Perez I. (2023). Characterization of a Piezoelectric Acoustic Sensor Fabricated for Low-Frequency Applications: A Comparative Study of Three Methods. Sensors.

[82] Fiorillo A., Critello C., Pullano S. (2018). Theory, Technology and Applications of Piezoresistive Sensors: A Review. Sens. Actuators A Phys..

[83] Algamili A.S., Khir M.H.M., Dennis J.O., Ahmed A.Y., Alabsi S.S., Ba Hashwan S.S., Junaid M.M. (2021). A Review of Actuation and Sensing Mechanisms in Mems-Based Sensor Devices. Nanoscale Res. Lett..

[84] Pan S., Zhang Z. (2019). Fundamental Theories and Basic Principles of Triboelectric Effect: A Review. Friction.

[85] Rathod V.T. (2020). A Review of Acoustic Impedance Matching Techniques for Piezoelectric Sensors and Transducers. Sensors.

[86] Li Z., Thong H.C., Zhang Y.F., Xu Z., Zhou Z., Liu Y.X., Cheng Y.Y.S., Wang S.H., Zhao C., Chen F. (2021). Defect Engineering in Lead Zirconate Titanate Ferroelectric Ceramic for Enhanced Electromechanical Transducer Efficiency. Adv. Funct. Mater..

[87] Rahaman A., Jung H., Kim B. (2021). Coupled D33 Mode-Based High Performing Bio-Inspired Piezoelectric Mems Directional Microphone. Appl. Sci..

[88] Yao Q., Xie L., Guo X., Yu F., Zhao X. (2023). Pvdf Membrane-Based Dual-Channel Acoustic Sensor Integrating the Fabry–Pérot and Piezoelectric Effects. Sensors.

[89] Qu M., Chen X., Yang D., Li D., Zhu K., Guo X., Xie J. (2021). Monitoring of Physiological Sounds with Wearable Device Based on Piezoelectric Mems Acoustic Sensor. J. Micromech. Microeng..

[90] Zhang Q., Wang Y., Li D., Xie J., Tao K., Hu P., Zhou J., Chang H., Fu Y. (2023). Multifunctional and Wearable Patches Based on Flexible Piezoelectric Acoustics for Integrated Sensing, Localization, and Underwater Communication. Adv. Funct. Mater..

[91] Han L., Liang W., Xie Q., Zhao J., Dong Y., Wang X., Lin L. (2023). Health Monitoring Via Heart, Breath, and Korotkoff Sounds by Wearable Piezoelectret Patches. Adv. Sci..

[92] Zhong Y., Wang Y., Ma L., He P., Qin J., Gao J., Li J. (2025). Ultrasensitive Piezoelectric Sensor Based on Polyimide Foam for Sound Recognition and Motion Monitoring. ACS Appl. Mater. Interfaces.

[93] Ma K., Chen H., Wu Z., Hao X., Yan G., Li W., Shao L., Meng G., Zhang W. (2022). A Wave-Confining Metasphere Beamforming Acoustic Sensor for Superior Human-Machine Voice Interaction. Sci. Adv..

[94] Zawawi S.A., Hamzah A.A., Majlis B.Y., Mohd-Yasin F. (2020). A Review of Mems Capacitive Microphones. Micromachines.

[95] Haque R.I., Loussert C., Sergent M., Benaben P., Boddaert X. (2015). Optimization of Capacitive Acoustic Resonant Sensor Using Numerical Simulation and Design of Experiment. Sensors.

[96] Rennoll V., McLane I., Eisape A., Grant D., Hahn H., Elhilali M., West J.E. (2023). Electrostatic Acoustic Sensor with an Impedance-Matched Diaphragm Characterized for Body Sound Monitoring. ACS Appl. Bio Mater..

[97] Zhang M., Wu G., Ren D., Gao R., Qi Z.M., Liang X. (2019). An Optical Mems Acoustic Sensor Based on Grating Interferometer. Sensors.

[98] Woo S., Han J.-H., Lee J.H., Cho S., Seong K.-W., Choi M., Cho J.-H. (2017). Realization of a High Sensitivity Microphone for a Hearing Aid Using a Graphene–Pmma Laminated Diaphragm. ACS Appl. Mater. Interfaces.

[99] Roh H., Kim D.H., Cho Y., Jo Y.M., Del Alamo J.A., Kulik H.J., Dinca M., Gumyusenge A. (2024). Robust Chemiresistive Behavior in Conductive Polymer/Mof Composites. Adv. Mater..

[100] Zhang S., Wang A., Cui S., Wang Z., Pan S., Wang R., Zhang W., Yilmaz M. (2025). Design and Acoustic Performance Study of Capacitive Acoustic Emission Sensors Based on Mems Technology. IEEE Sens. J..

[101] Zhou C., Zang J., Xue C., Ma Y., Hua X., Gao R., Zhang Z., Li B., Zhang Z. (2022). Design of a Novel Medical Acoustic Sensor Based on Mems Bionic Fish Ear Structure. Micromachines.

[102] Duanmu Z., Kong C., Guo Y., Zhang X., Liu H., Zhao C., Gong X., Cai C., Ho C., Wan C. (2022). Design and Implementation of an Acoustic-Vibration Capacitive Mems Microphone. AIP Adv..

[103] Li Y., Li Y., Zhang R., Li S., Liu Z., Zhang J., Fu Y. (2023). Progress in Wearable Acoustical Sensors for Diagnostic Applications. Biosens. Bioelectron..

[104] Chung D.D.L. (2020). A Critical Review of Piezoresistivit and Its Application in Electrical-Resistance-Based Strain Sensing. J. Mater. Sci..

[105] Utzeri M., Cebeci H., Kumar S. (2025). Autonomous Sensing Architected Materials. Adv. Funct. Mater..

[106] Irani F.S., Shafaghi A.H., Tasdelen M.C., Delipinar T., Kaya C.E., Yapici G.G., Yapici M.K. (2022). Graphene as a Piezoresistive Material in Strain Sensing Applications. Micromachines.

[107] Shiri M., Nouri N.M., Riahi M. (2025). Design and Fabrication of a Leg Based Stretchable Piezoresistive Acoustic Pressure Sensor for Ultra Low Pressures. Sci. Rep..

[108] Le T.S.D., Phan H.P., Kwon S., Park S., Jung Y., Min J., Chun B.J., Yoon H., Ko S.H., Kim S.W. (2022). Recent Advances in Laser-Induced Graphene: Mechanism, Fabrication, Properties, and Applications in Flexible Electronics. Adv. Funct. Mater..

[109] Vivaldi F.M., Dallinger A., Bonini A., Poma N., Sembranti L., Biagini D., Salvo P., Greco F., Di Francesco F. (2021). Three-Dimensional (3D) Laser-Induced Graphene: Structure, Properties, and Application to Chemical Sensing. ACS Appl. Mater. Interfaces.

[110] Mensah S.A., El-Bab A.M.F., Tominaga Y., Khalil A.S. (2025). Precisely Engineered Interface of Laser-Induced Graphene and Mos2 Nanosheets for Enhanced Supercapacitor Electrode Performance. Appl. Surf. Sci..

[111] Liu H., Dong M., Huang W., Gao J., Dai K., Guo J., Zheng G., Liu C., Shen C., Guo Z. (2017). Lightweight Conductive Graphene/Thermoplastic Polyurethane Foams with Ultrahigh Compressibility for Piezoresistive Sensing. J. Mater. Chem. C.

[112] Chen S., Zhang P., Zhao J., Novoselov K.S., Andreeva D.V. (2025). Graphene Oxide/DNA-Aerogel Pressure and Acoustic Sensor. Nanoscale Horiz..

[113] Ye R., James D.K., Tour J.M. (2019). Laser-Induced Graphene: From Discovery to Translation. Adv. Mater..

[114] Sun Q.J., Zhuang J., Venkatesh S., Zhou Y., Han S.T., Wu W., Kong K.W., Li W.J., Chen X., Li R.K.Y. (2018). Highly Sensitive and Ultrastable Skin Sensors for Biopressure and Bioforce Measurements Based on Hierarchical Microstructures. ACS Appl. Mater. Interfaces.

[115] Okamoto Y., Nguyen T.V., Takahashi H., Takei Y., Okada H., Ichiki M. (2023). Highly Sensitive Low-Frequency-Detectable Acoustic Sensor Using a Piezoresistive Cantilever for Health Monitoring Applications. Sci. Rep..

[116] Wang X., Tang Y., Cheng S., Gao Q., Yuan Y., Li A., Guan S. (2022). Pdms-Based Conductive Elastomeric Composite with 3d Reduced Graphene Oxide Conductive Network for Flexible Strain Sensor. Compos. Part A Appl. Sci. Manuf..

[117] Yang J., Chen J., Liu Y., Yang W., Su Y., Wang Z.L. (2014). Triboelectrification-Based Organic Film Nanogenerator for Acoustic Energy Harvesting and Self-Powered Active Acoustic Sensing. ACS Nano.

[118] Wu P., Wang F., Xu S., Liu T., Qi Y., Zhao X., Zhang C., Mu X. (2023). A Highly Sensitive Triboelectric Quasi-Zero Stiffness Vibration Sensor with Ultrawide Frequency Response. Adv. Sci..

[119] Kim I., Cho H., Kim D. (2024). Frequency Detection for String Instruments Using 1d-2d Non-Contact Mode Triboelectric Sensors. Micromachines.

[120] Sun W., Chen J., Yuan T., Sui D., Zhou J. (2024). A Novel Tiny Triboelectric Acoustic Sensor Design Based on Nanocomposite Enhancement for Highly-Sensitive, Broadband, and Self-Powered Multi-Functional Applications. Nano Energy.

[121] Chang H., Zhao J., Qin R., Bao W., Xie H., Tan Y., Guo Z., Zou H., Wang X., Dong K. (2025). Ultra-Wideband Hybrid Triboelectric–Piezoelectric Acoustic Sensors Enabled by Acoustic Metasurface Lens for Environment Perception and Medical Imaging. Adv. Funct. Mater..

[122] Park J., Kang D.H., Chae H., Ghosh S.K., Jeong C., Park Y., Cho S., Lee Y., Kim J., Ko Y. (2022). Frequency-Selective Acoustic and Haptic Smart Skin for Dual-Mode Dynamic/Static Human-Machine Interface. Sci. Adv..

[123] Shanbedi M., Ardebili H., Karim A. (2023). Polymer-Based Triboelectric Nanogenerators: Materials, Characterization, and Applications. Progress. Polym. Sci..

[124] Li G.Z., Wang G.G., Ye D.M., Zhang X.W., Lin Z.Q., Zhou H.L., Li F., Wang B.L., Han J.C. (2019). High-Performance Transparent and Flexible Triboelectric Nanogenerators Based on Pdms-Ptfe Composite Films. Adv. Electron. Mater..

[125] Lee K., Mhin S., Han H., Kwon O., Kim W.-B., Song T., Kang S., Kim K.M. (2022). A High-Performance Pdms-Based Triboelectric Nanogenerator Fabricated Using Surface-Modified Carbon Nanotubes Via Pulsed Laser Ablation. J. Mater. Chem. A.

[126] Koç M., Tatardar F., Musayeva N.N., Guluzade S., Sarı A., Paralı L. (2024). The Piezoelectric Properties of Three-Phase Electrospun Pvdf/Pzt/Multiwalled Carbon Nanotube Composites for Energy Harvesting Applications. J. Alloys Compd..

[127] Huang A., Zhu Y., Peng S., Tan B., Peng X. (2024). Improved Energy Harvesting Ability of Single-Layer Binary Fiber Nanocomposite Membrane for Multifunctional Wearable Hybrid Piezoelectric and Triboelectric Nanogenerator and Self-Powered Sensors. ACS Nano.

[128] Esteves D.S., Pereira M.F.C., Ribeiro A., Duraes N., Paiva M.C., Sequeiros E.W. (2023). Development of Mwcnt/Magnetite Flexible Triboelectric Sensors by Magnetic Patterning. Polymers.

[129] Sasmal A., Patra A., Arockiarajan A. (2022). Tuning the Space Charge Polarization of Pvdf Based Ternary Composite for Piezo-Tribo Hybrid Energy Harvesting. Appl. Phys. Lett..

[130] Niu S., Wang Z.L. (2015). Theoretical Systems of Triboelectric Nanogenerators. Nano Energy.

[131] Hui X., Tang L., Zhang D., Yan S., Li D., Chen J., Wu F., Wang Z.L., Guo H. (2024). Acoustically Enhanced Triboelectric Stethoscope for Ultrasensitive Cardiac Sounds Sensing and Disease Diagnosis. Adv. Mater..

[132] Xia K., Xu Z. (2021). Applying a Triboelectric Nanogenerator by Using Facial Mask for Flexible Touch Sensor. Sens. Actuators A Phys..

[133] Shahbaz I., Yao Z., Rehman S.U., Deng J., Song Y., Li L. (2025). Self-Powered Triboelectric–Piezoelectric Hybrid Acoustic Sensor with Microcone Patterned Film Based on Poly (Vinylidene Fluoride)/Lnknts-Mn Nanorods Composite. Clin. Med. Res..

[134] Babu A., Malik P., Das N., Mandal D. (2022). Surface Potential Tuned Single Active Material Comprised Triboelectric Nanogenerator for a High Performance Voice Recognition Sensor. Small.

[135] Tat T., Libanori A., Au C., Yau A., Chen J. (2021). Advances in Triboelectric Nanogenerators for Biomedical Sensing. Biosens. Bioelectron..

[136] Pu X., An S., Tang Q., Guo H., Hu C. (2021). Wearable Triboelectric Sensors for Biomedical Monitoring and Human-Machine Interface. iScience.

[137] Pareek D., Islam S.M., Singh J. (2024). Bulk Acoustic Wave Resonators for Sensing Applications: A Review. Sens. Actuators A Phys..

[138] Russell D.A. (2021). Using Impedance to Explore Resonance in Mechanical, Acoustical, and Electrical Analog Systems. J. Acoust. Soc. Am..

[139] Fogel R., Limson J., Seshia A.A. (2016). Acoustic Biosensors. Essays Biochem..

[140] Yang Y., Vallecchi A., Shamonina E., Stevens C.J., You Z. (2023). A New Class of Transformable Kirigami Metamaterials for Reconfigurable Electromagnetic Systems. Sci. Rep..

[141] Hwang J., Hong S. (2025). Passive Frequency Tunability in Moiré-Inspired Frequency Selective Surfaces Based on Full-Wave Simulation. Micromachines.

[142] Zhang W.M., Hu K.M., Peng Z.K., Meng G. (2015). Tunable Micro- and Nanomechanical Resonators. Sensors.

[143] Lu K., Wu K., Li Q., Zhou X., Zhang Y., Xi X., Wu X., Xiao D. (2022). Dispersive Resonance Modulation Based on the Mode-Coupling Effect in a Capacitive Micromechanical Resonator. Phys. Rev. Appl..

[144] Nastro A., Ferrari M., Rufer L., Basrour S., Ferrari V. (2022). Piezoelectric MEMS Acoustic Transducer with Electrically-Tunable Resonant Frequency. Micromachines.

[145] Wang Y., Zou Y., Gao C., Gu X., Ma Y., Liu Y., Liu W., Soon J.B.W., Cai Y., Sun C. (2022). Effects of Electric Bias on Different Sc-Doped Aln-Based Film Bulk Acoustic Resonators. Electronics.

[146] Cha J., Daraio C. (2018). Electrical Tuning of Elastic Wave Propagation in Nanomechanical Lattices at Mhz Frequencies. Nat. Nanotechnol..

[147] Jeon N., Noh J., Jung C., Rho J. (2022). Electrically Tunable Metasurfaces: From Direct to Indirect Mechanisms. New J. Phys..

[148] Pang W., Zhang H., Yu H., Lee C.-Y., Kim E.S. (2007). Electrical Frequency Tuning of Film Bulk Acoustic Resonator. J. Microelectromech. Syst..

[149] Kanj A., Ferrari P., van der Zande A.M., Vakakis A.F., Tawfick S. (2023). Ultra-Tuning of Nonlinear Drumhead Mems Resonators by Electro-Thermoelastic Buckling. Mech. Syst. Signal Process..

[150] Branch D.W., Jensen D.S., Nordquist C.D., Siddiqui A., Douglas J.K., Eichenfield M., Friedmann T.A. (2020). Investigation of a Solid-State Tuning Behavior in Lithium Niobate. IEEE Trans. Ultrason. Ferroelectr. Freq. Control.

[151] Nastro A., Rufer L., Ferrari M., Basrour S., Ferrari V. Piezoelectric Micromachined Acoustic Transducer with Electrically-Tunable Resonant Frequency. Proceedings of the 2019 20th International Conference on Solid-State Sensors, Actuators and Microsystems & Eurosensors XXXIII (TRANSDUCERS & EUROSENSORS XXXIII).

[152] Koutserimpas T.T., Rivet E., Lissek H., Fleury R. (2019). Active Acoustic Resonators with Reconfigurable Resonance Frequency, Absorption, and Bandwidth. Phys. Rev. Appl..

[153] Guo X., Volery M., Lissek H. (2022). Pid-Like Active Impedance Control for Electroacoustic Resonators to Design Tunable Single-Degree-of-Freedom Sound Absorbers. J. Sound. Vib..

[154] Allen W.N., Gao A., Gong S., Peroulis D. Simultaneous Analog Tuning of the Series-and Anti-Resonances of Acoustic Wave Resonators. Proceedings of the 2018 IEEE 19th Wireless and Microwave Technology Conference (WAMICON).

[155] Zhang Y., Wu K., Zhang X., Liu X., Huang L. (2022). A Programmable Resonator Based on a Shunt-Electro-Mechanical Diaphragm. Int. J. Mech. Sci..

[156] Arora D., Kaur D. (2024). Magnetically and Electrically Tunable Pb (Mg1/3nb2/3) O3–Pbtio3/Ferromagnetic Shape Memory Alloy Nanolayered Film-Based Acoustic Wave Resonator for Flexible Microelectromechanical Systems. ACS Appl. Nano Mater..

[157] Sun J., Cai W., Yang Y., Zhuang Y., Zhang Q. (2025). 2D α-In2Se3 Flakes for High Frequency Tunable and Switchable Film Bulk Acoustic Wave Resonators. Adv. Electron. Mater..

[158] Mujahid A., Dickert F.L. (2017). Surface Acoustic Wave (Saw) for Chemical Sensing Applications of Recognition Layers. Sensors.

[159] de Oliveira E.R.C., Vensaus P., Soler-Illia G.J., Lanzillotti-Kimura N.D. (2023). Design of Cost-Effective Nanoacoustic Devices Based on Mesoporous Thin Films. arXiv.

[160] Gardiner A., Domingo-Roca R., Windmill J.F.C., Feeney A. (2024). An Adjustable Acoustic Metamaterial Cell Using a Magnetic Membrane for Tunable Resonance. Sci. Rep..

[161] Arora D., Shankhdhar S., Burman T., Singh J., Kaur D. (2025). Magnetically and Electrically Tunable Pb (Mg 1/3 Nb 2/3) O 3–Pbtio 3 and Ni-Mn-in Based Surface Acoustic Wave Resonator. IEEE Sens. J..

[162] Zhang W., Xin F. (2024). Broadband Low-Frequency Sound Absorption Via Helmholtz Resonators with Porous Material Lining. J. Sound. Vib..

[163] Ni X., Chen K., Weiner M., Apigo D.J., Prodan C., Alu A., Prodan E., Khanikaev A.B. (2019). Observation of Hofstadter Butterfly and Topological Edge States in Reconfigurable Quasi-Periodic Acoustic Crystals. Commun. Phys..

[164] Chong X.-Y., Chen A.-L., Du X.-Y., Zhao S.-D., Wang Y.-S. (2025). Design of the Reconfigurable Rotationally Symmetric Resonant Functional Unit and Curved Metasurface for Acoustic Wavefront Modulation. Compos. Struct..

[165] Lee S., Kim W., Park N.C., Park J.W. (2023). Frequency Selectivity Via Inner Boundary Conditions for a Self-Powered Multiresonant Acoustic Sensing Array with Broad Bandwidth. Adv. Funct. Mater..

[166] Liu C., Dong P., Wang J., Deng Z., Luo J., Liu C., Wu J., Tan K., Pan J., Han R. (2025). Frog Vocal Sacs-Inspired Soft Acoustic System with Continuously Tunable Resonance for Sound Emission and Stethoscopic Sensing. Sci. Adv..

[167] Wei Y.H., Guo Z.F., Wang Y.F., Lin T., Hou W.W., Duan S.W., Tao L.Q., Tian H., Yang Y., Ren T.L. (2025). Frequency-Tunable Sound Amplification in a Conch-Like Cavity with Graphene Thermoacoustic Resonance. Sci. Adv..

[168] Cheng H., Yang F., Shen X., Yang X., Zhang X., Bi S. (2023). Study on a Hexagonal Acoustic Metamaterial Cell of Multiple Parallel-Connection Resonators with Tunable Perforating Rate. Materials.

[169] Liu S., Chen F. (2025). Dynamically Tunable Fano Resonance Effect Based on Monolayer Graphene with Disk Defect Robustness. Phys. B Condens. Matter.

[170] Wang A., Sahandabadi S., Harrison T., Spicer D., Ahamed M.J. (2022). Modelling of Air Damping Effect on the Performance of Encapsulated Mems Resonators. Microsyst. Technol..

[171] Kong F., Zou Y., Li Z., Deng Y. (2024). Advances in Portable and Wearable Acoustic Sensing Devices for Human Health Monitoring. Sensors.

[172] Hallil H., Dejous C., Hage-Ali S., Elmazria O., Rossignol J., Stuerga D., Talbi A., Mazzamurro A., Joubert P.-Y., Lefeuvre E. (2021). Passive Resonant Sensors: Trends and Future Prospects. IEEE Sens. J..

[173] Liu Z., Qu K., Chen K., Li Z. (2024). Multi-Type Stochastic Resonances for Noise-Enhanced Mechanical, Optical, and Acoustic Sensing. Research.

[174] Ivancic J., Karunasiri G., Alves F. (2023). Directional Resonant MEMS Acoustic Sensor and Associated Acoustic Vector Sensor. Sensors.

[175] Cuong V.N., Bui C.M. (2025). Development of an Electrostatic Tuning Method for Resonant Frequency Control in a Single-Axis Mems Accelerometer. EUREKA Phys. Eng..

[176] Leo A., Bramanti A.P., Giusti D., Quaglia F., Maruccio G. (2023). Reconfigurable Split Ring Resonators by Mems-Driven Geometrical Tuning. Sensors.

[177] Yang Y., Peng B., Yue H., Huang F., He P., He Z., Zhu J., Zhang W. (2022). Temperature Characteristics of Surface Acoustic Wave Resonators Prepared on (0, 90, Ψ) Ctgs Cuts. Appl. Acoust..

[178] Panah D.S., Hines A., McKeever S. (2023). Exploring the Impact of Noise and Degradations on Heart Sound Classification Models. Biomed. Signal Process. Control.

[179] Gubbi V., Ivanov T., Ved K., Lenk C., Ziegler M. (2025). Bio-Inspired Acoustic Mems Sensor with Tunable Resonance Frequency. Sens. Actuators A Phys..

